# Quartet RNA reference materials improve the quality of transcriptomic data through ratio-based profiling

**DOI:** 10.1038/s41587-023-01867-9

**Published:** 2023-09-07

**Authors:** Ying Yu, Wanwan Hou, Yaqing Liu, Haiyan Wang, Lianhua Dong, Yuanbang Mai, Qingwang Chen, Zhihui Li, Shanyue Sun, Jingcheng Yang, Zehui Cao, Peipei Zhang, Yi Zi, Ruimei Liu, Jian Gao, Naixin Zhang, Jingjing Li, Luyao Ren, He Jiang, Jun Shang, Sibo Zhu, Xiaolin Wang, Tao Qing, Ding Bao, Bingying Li, Bin Li, Chen Suo, Yan Pi, Xia Wang, Fangping Dai, Andreas Scherer, Pirkko Mattila, Jinxiong Han, Lijun Zhang, Hui Jiang, Danielle Thierry-Mieg, Jean Thierry-Mieg, Wenming Xiao, Huixiao Hong, Weida Tong, Jing Wang, Jinming Li, Xiang Fang, Li Jin, Joshua Xu, Feng Qian, Rui Zhang, Leming Shi, Yuanting Zheng

**Affiliations:** 1 State Key Laboratory of Genetic Engineering, School of Life Sciences and Human Phenome Institute, Shanghai Cancer Center, Fudan University, Shanghai, China; 2 National Institute of Metrology, Beijing, China; 3 Greater Bay Area Institute of Precision Medicine, Guangzhou, China; 4 Nextomics Biosciences Institute, Wuhan, China; 5 Genome Decoding Institute, Nantong, China; 6 Institute for Molecular Medicine Finland (FIMM), University of Helsinki, Helsinki, Finland; 7 EATRIS ERIC-European Infrastructure for Translational Medicine, Amsterdam, The Netherlands; 8 Nanjing Vazyme Biotech Co. Ltd., Nanjing, China; 9 MGI, BGI-Shenzhen, Shenzhen, China; 10 National Center for Biotechnology Information, National Library of Medicine, National Institutes of Health, Bethesda, MD USA; 11 Office of Oncologic Diseases, Office of New Drugs, Center for Drug Evaluation and Research, US Food and Drug Administration, Silver Spring, MD USA; 12 Division of Bioinformatics and Biostatistics, National Center for Toxicological Research, US Food and Drug Administration, Jefferson, AR USA; 13 National Center for Clinical Laboratories, Institute of Geriatric Medicine, Chinese Academy of Medical Sciences, Beijing Hospital, Beijing, China; 14 National Center of Gerontology, Beijing, China; 15 Shanghai Public Health Clinical Center, Fudan University, Shanghai, China; 16 International Human Phenome Institutes, Shanghai, China

**Keywords:** Quality control, Standardization, Transcriptomics

## Abstract

Certified RNA reference materials are indispensable for assessing the reliability of RNA sequencing to detect intrinsically small biological differences in clinical settings, such as molecular subtyping of diseases. As part of the Quartet Project for quality control and data integration of multi-omics profiling, we established four RNA reference materials derived from immortalized B-lymphoblastoid cell lines from four members of a monozygotic twin family. Additionally, we constructed ratio-based transcriptome-wide reference datasets between two samples, providing cross-platform and cross-laboratory ‘ground truth’. Investigation of the intrinsically subtle biological differences among the Quartet samples enables sensitive assessment of cross-batch integration of transcriptomic measurements at the ratio level. The Quartet RNA reference materials, combined with the ratio-based reference datasets, can serve as unique resources for assessing and improving the quality of transcriptomic data in clinical and biological settings.

## Abstract (web summary)

A new RNA reference set improves detection of differential expression in clinical settings.

## Main

RNA sequencing (RNA-seq) is an indispensable tool for transcriptome-wide analysis of differential gene expression and is widely used in biomedical research to discover biomarkers for clinical diagnosis, prognosis and therapeutic action^1–5^. As transcriptome-based biomarker discovery continues to advance, RNA-seq-based assays will routinely be used within the clinic^3,6,7^. For example, clinical tests complemented by measuring the differential expression of clinically relevant genes will facilitate the prediction of clinical outcomes and treatment decisions^8–10^. It should be noticed that clinically relevant differences in gene expression among study groups are often small^11–13^. Hence, there is a consistent need for making RNA-seq more reliable to enhance its power of detecting subtle differential expression, especially for clinical applications such as companion diagnostics and prognostics. The reliability of RNA-seq technology comprises two aspects. It must be ensured that data from a certain laboratory or batch are acquired with the best proficiency obtainable with the technology (intra-batch)^14^, and similar differential expression results from replicate samples processed with different platforms, laboratories, protocols or batches should be required (cross-batch)^15^. Cross-batch reproducibility also refers to multi-batch integrability, which is the ability to provide similar results between within-batch analysis and cross-batch integrative analysis in the existence of widespread batch effects^16,17^.

Reference materials are valuable tools for evaluating the reliability of omic data^18,19^. Based on RNA-seq data generated with reference materials from different platforms, laboratories or batches, reliability can be objectively evaluated according to the two aforementioned aspects of intra-batch (or laboratory) proficiency and cross-batch reproducibility. The MicroArray/Sequencing Quality Control (MAQC/SEQC) consortia previously established two publicly available transcriptome-wide RNA reference materials that are derived from 10 cancer cell lines and brain tissues of 23 donors^15^. Based on these RNA reference materials, the MAQC/SEQC consortia systematically evaluated the performances of different platforms and laboratories in using the microarray^15^ and RNA-seq^20,21^ technologies, which have served as resources for the research community to develop and validate new RNA quantification technologies^22^.

However, the ability to successfully distinguish the two MAQC RNA reference materials does not guarantee that the underlying transcriptomic profiling system can be used to detect subtle differential expression for clinical diagnosis purposes. First, the considerable biological differences between the two MAQC reference materials^23^ are substantially greater than groupwise differences commonly seen in most clinically relevant scenarios. Second, the ability of distinguishing two MAQC RNA sample groups does not translate to the ability of reliably distinguishing more than two sample groups as commonly seen in clinical applications. Third, the current stock of the MAQC B sample is almost exhausted^24^, and it is difficult to be regenerated. Therefore, there is an urgent need for a multiple-group RNA reference materials suite with subtle inter-sample differences, high stability, long-term availability and easy manufacturability.

Furthermore, reference datasets can be used as ‘ground truth’ in performance assessment. Previous studies have shown that genome-wide reference datasets of genetic variants enable improvement of the reproducibility and accuracy of clinical applications of cancer^25–27^ and genetic diseases^28–30^. However, there is a paucity of transcriptome-wide reference datasets^3,18^. Therefore, transcriptome-wide reference datasets associated with publicly available RNA reference materials are urgently needed but are lacking^18^.

As a part of the Quartet Project for the quality control and data integration of multi-omics profiling (http://chinese-quartet.org/), we established four RNA reference materials derived from immortalized B-lymphoblastoid cell lines (LCLs) from the four members of a monozygotic twin family quartet, which exhibited subtle inter-sample differences, high stability, long-term availability and easy manufacturability. Furthermore, matched multi-omics reference materials, including DNAs^31^, proteins^32^ and metabolites^33^, were established along with RNAs from the same culturing of the LCLs to enable integrative omics analyses. In this study, we performed a multi-laboratory RNA-seq study based on 21 batches of multi-laboratory RNA-seq datasets generated with different protocols, established ratio-based reference datasets of gene expression and developed quality metrics for assessing reliability of RNA-seq technology in terms of intra-batch proficiency and cross-batch reproducibility.

## Results

### Overview of study design

**Fig. 1:**
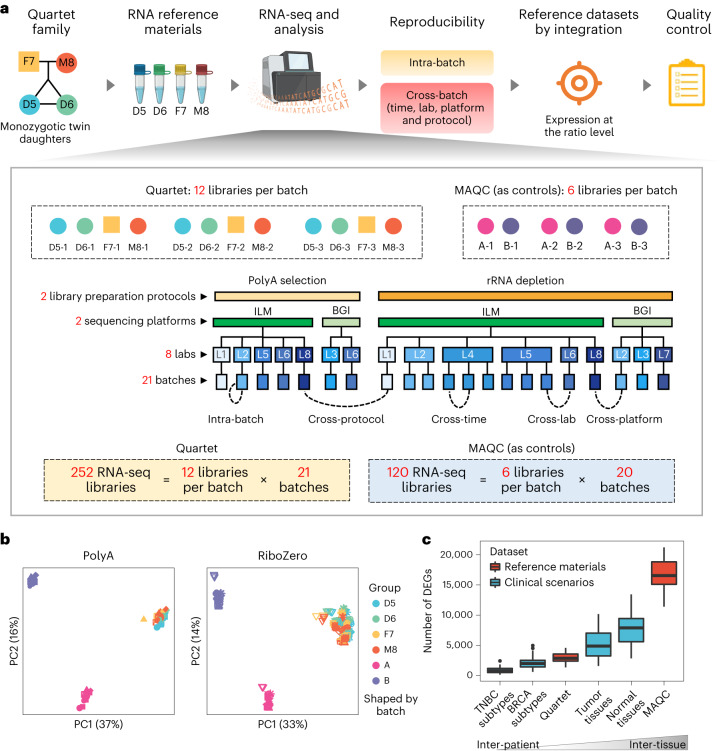
Overview of study design. **a**, Quartet RNA reference materials were derived from immortalized EBV-infected B-LCLs from a quartet family, including monozygotic twin daughters (D5 and D6) and their father (F7) and mother (M8). Multi-batches of RNA-seq datasets were generated from independent laboratories using different library preparation protocols and sequencing platforms. Intra-batch proficiency and cross-batch reproducibility were then estimated. Based on multi-batches of RNA-seq data, we constructed ratio-based transcriptome-wide reference datasets and developed corresponding quality metrics. **b**, Scatter plots of PCs on RNA-seq data of the Quartet and MAQC RNA reference materials (marked in colors) across 20 batches (marked in shapes; see Supplementary Fig. 4 for details). log_2_-transformed FPKM values were used for PCA. **c**, Box plots showing the numbers of DEGs among Quartet reference materials, MAQC reference materials and four clinical/biological classification problems from published datasets. The four clinical/biological classifications used to represent clinical scenarios include four subtypes of TNBCs with different therapeutic actions (basal-like and immune-suppressed, luminal androgen receptor, immunomodulatory subtype and mesenchymal-like subtypes)^34^, four subtypes of breast cancers (BRCAs) with different prognosis and therapeutic actions (luminal A, luminal B, basal-like/triple negative and HER2-positive subtypes)^35^, four types of tumor tissues (brain, breast, kidney and lung cancers)^35^ and four types of normal tissues (brain, breast, kidney and lung)^36^. The latter two types of biological classification problems are important for understanding the genetic basis of human diseases. Three samples from each clinical subtype or biological group were randomly selected for differential expression analysis to eliminate effect of number of samples used for analysis. A gene was identified as differentially expressed when satisfying the criteria of Student’s *t*-test two-sided *P* < 0.05 and fold change ≥2 or ≤0.5 between two groups or conditions. To eliminate selection biases, this process was repeated 20 times (*n* = 20). The box plots display the distribution of data with the median represented by the line inside the box and the interquartile range represented by the box. The whiskers extend from the box to the minimum and maximum values that are not outliers.

The Quartet RNA reference materials were derived from the Epstein–Barr virus (EBV) LCLs from four members of a Chinese family quartet, including monozygotic twin daughters (D5 and D6), father (F7) and mother (M8) (Fig. 1a). They have been certified by China’s State Administration for Market Regulation as the First Class of National Reference Materials and are extensively being used for proficiency testing and method validation. The certified reference material numbers are GBW09904 (D5), GBW09905 (D6), GBW09906 (F7) and GBW09907 (M8).

Large quantities of RNA (over 5 mg) were obtained per cell line, enabling standard RNA-seq experiments over 10,000 to 50,000 times and providing a material basis for long-term quality monitoring. RNA quality was high according to RNA integrity number (RIN) and RNA purity (Supplementary Fig. 1 and Supplementary Table 1). Moreover, the RNA reference materials showed adequate stability across 20 months of storage at −80 °C or 14 d of storage at room temperature (25 °C) or 4 °C or up to 20 times of bottle-opening and freeze–thaw cycle (Supplementary Fig. 2).

RNA-seq datasets from the Quartet RNA reference materials were then collected, consisting of 252 RNA-seq libraries from 21 batches generated in eight laboratories using two library construction protocols (PolyA selection and RiboZero) and two sequencing platforms (Illumina NovaSeq (ILM) and MGI DNBSEQ-T7 (BGI)) (Fig. 1a and Supplementary Table 2). Here, a batch is defined as 12 libraries from a standard sample set, consisting of 12 vials with each representing one of the triplicates of the Quartet RNA reference sample groups, whose library construction and sequencing experiments were conducted simultaneously. On the other hand, libraries constructed at different timepoints, in different laboratories, with different sequencing platforms, or using different library preparation protocols, are recognized broadly as cross-batch libraries (Fig. 1a). This comprehensive study design allows for objective performance assessment at multiple levels, including cross-time, cross-laboratory, cross-platform and cross-protocol. Moreover, RNA-seq experiments with the MAQC RNA reference materials (A and B) were conducted simultaneously with the Quartet reference materials in 20 of the 21 batches (Fig. 1a), enabling head-to-head comparisons between the two sources (MAQC versus Quartet) of RNA reference materials. In addition, the bioinformatic analysis pipeline was validated using published data from the MAQC RNA reference materials by comparison with previous studies^15,20^ (Supplementary Fig. 3).

### The Quartet exhibits small intrinsic biological differences

Using principal component analysis (PCA) as an exploratory overview of data analysis, we found that multi-batch libraries of the Quartet reference materials from the same protocol (PolyA or RiboZero) were clustered together, whereas libraries of MAQC A and B samples were clustered separately into distinct groups according to protocol and sample groups (Fig. 1b and Supplementary Fig. 4). This result indicates that the intrinsic biological differences among the four groups of Quartet RNA reference materials are much smaller compared to those between the two MAQC RNA reference materials.

To investigate whether the magnitude of intrinsic biological differences or signals between the Quartet reference materials is representative of those seen in clinically relevant scenarios, we compared the extent of intrinsic biological differences between reference materials (MAQC A versus B and Quartet members) and those of four biological classification problems from published datasets ranging from four subtypes of triple-negative breast cancers (TNBCs)^34^, four subtypes of breast cancers^35^, four types of tumor tissues^35^ and four types of normal tissues^36^. The number of differentially expressed genes (DEGs), previously used as a measure of ‘treatment effect size’^13^, identified from the four biological classification problems ranged from 884 to 4,980 (mean), corresponding to an increase of intrinsic biological differences and/or decrease of within-group heterogeneity (Fig. 1c). Notably, the differences among Quartet RNA reference materials were 2,164 (mean) in terms of DEGs, which were ranked in the middle of these four clinical classification scenarios. In contrast, the differences between the two MAQC RNA reference materials were much larger (16,503, mean) than those observed in the aforementioned biological classification problems (Fig. 1c). These data again illustrate that the intrinsic biological differences among the Quartet reference materials are much smaller than those between MAQC RNA reference materials A and B and that such small differences are similar to those seen in clinical and biological classification scenarios.

### Signal-to-noise ratio enables assessment of data quality

**Fig. 2:**
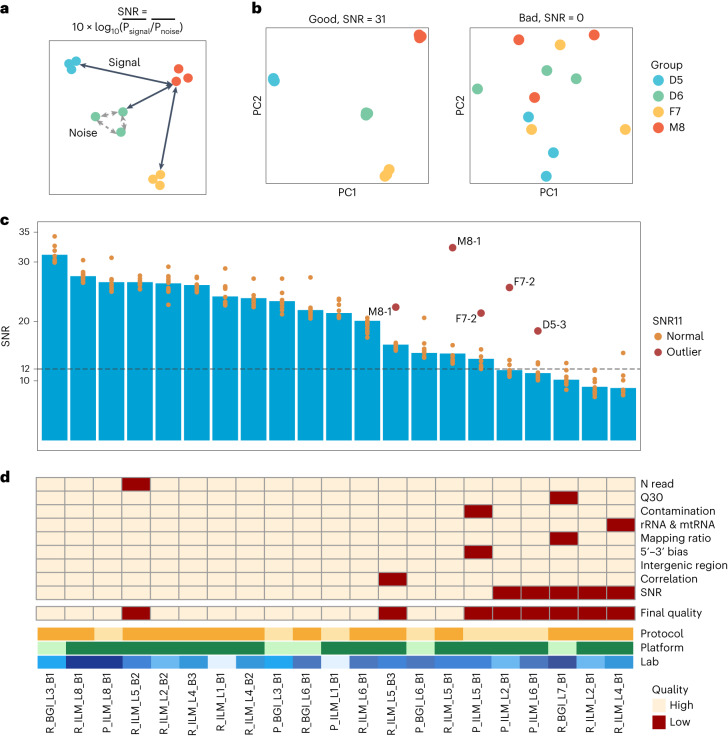
SNR enables assessment and diagnosis of data quality. **a**, Concept of calculating SNR. SNR was established to characterize the ability of a platform, a laboratory or a batch to distinguish the intrinsic differences among distinct biological sample groups (‘signal’) from variations in technical replicates of the same sample group (‘noise’). **b**, Examples of good and bad batches with their SNR values and corresponding PCA scatter plots. **c**, SNR values across 21 RNA-seq batches to measure data quality. Batches were ordered by SNR values. Dots represent SNR values based on any 11 of the 12 libraries (SNR11) in each batch. A dot in dark red represents SNR11 value that increased over 6 dB compared to its standard SNR (12-sample SNR), when one library in this batch was excluded (the library ID was labeled), whereas a dot in orange represents SNR11 value that decreased or increased less than 6 dB compared to its standard SNR. **d**, Quality flags of RNA-seq batches in terms of the number of sequencing reads (N read), percentage of Q30 (Q30), percentage of reads that were mapped to contamination species (for example, virus, bacteria and fungi) (Contamination), percentage of reads that were mapped to rRNA or mtRNA (rRNA & mtRNA), percentage of reads that were mapped to the human genome (Mapping ratio), gene body (5′–3′) bias (5′–3′ bias), percentage of mapped reads that were located in intergenic region in human genome (Intergenic region), Pearson correlation coefficient of technical replicates (Correlation), SNR and Final quality flag. Batches were ordered by SNR values. Protocol, Platform and Lab information of each batch is shown by the color legend.

Based on the Quartet design, a signal-to-noise ratio (SNR) metric was established to gauge the performance of a platform, a laboratory, a protocol or a batch in distinguishing the intrinsic biological differences (‘signal’) among the Quartet samples from variations among technical replicates of the same sample group (‘noise’) (Fig. 2a). Generally, a lower SNR value indicates lower discriminating power and vice versa. An SNR value around or below zero means that the magnitude of signal is at a similar level as the noise or even lower than the noise. In this case, it is impossible to distinguish different sample groups under the high level of technical noises (Fig. 2b).

We evaluated the performance of five different methods in defining the SNR depending on whether the calculation is based on the original feature space or the dimensionality-reduced space (see Methods for details), including OriAll_EucDist, OriSingle_MedianEucDist, OriAll_1-Cor, ReducedDim_tSNE and ReducedDim_PCA. It was found that the PCA-based SNR outperformed the other four methods in terms of its sensitivity in differentiating the quality of different datasets, as seen by a larger variability and a higher value of the SNR (Supplementary Fig. 5a). We next computed SNR using different numbers of principal components (PCs) of PCA (Supplementary Fig. 5b). SNR values based on the first component, the first two components or the first three components were highly correlated (Supplementary Fig. 5b). With the desire to maximize the range (that is, variability) of SNR over experiment batches and to match the good visual presentation of batch quality control, we chose SNR computed with the first two PCs.

SNR enables assessment of quality across the 21 batches of RNA-seq data. For most batches, the three replicates from the same sample group can be clearly distinguished from those of other sample groups (Supplementary Fig. 6). Large fluctuations of SNR values were observed across batches generated with the same protocol, the same sequencing platform or even from the same laboratory, highlighting the need for objectively assessing and monitoring the technical competency in data generation (Fig. 2c). Using an SNR cutoff of 12 (mean − s.d. across 21 batches), batches were flagged as high and low quality (Fig. 2d). It should be noted that SNR values based on different bioinformatics pipelines might differ, whereas the trend of the SNR across batches remained similar (Supplementary Fig. 7).

SNR can also be applied to diagnose potential causes of quality issues. In addition to the SNR values considering all 12 libraries in a batch, we also calculated SNR11 values with any 11 of the 12 libraries in each batch (Fig. 2c). In five batches, the SNR11 values increased by more than 6 dB compared to the corresponding 12-sample SNR values, indicating that the lower SNR values from these five batches might be a result of a ‘random failure’ of a particular technical replicate (for example, replicate M8-1 from batch L5_B1 and replicate F7-2 from batch L2_B1). In contrast, the three batches with the lowest SNR values were possibly due to systematic technical issues, because excluding any specific replicate (or potential outlier) could not greatly improve the SNR values.

Moreover, SNR enables assessment of data quality not only at gene expression level but also at alternative splicing (AS) level. Similarly, SNR values at AS level varied across batches. SNR values could be as high as 32.3, so that the three technical replicates for each sample type on the PCA plot could be loosely regarded as one dot (Supplementary Fig. 8a) or as low as 2.4 where technical replicates of one sample type were mixed with libraries from other sample types (Supplementary Fig. 8b).

Using multiple metrics, including SNR and other widely used quality metrics, with fastq, bam and expression profiles, with SNR showing the greatest differentiating power, 13 batches were flagged as high quality and were used for subsequent data integration to create the reference datasets, whereas the other eight batches were flagged as low quality and excluded from constructing the reference datasets (Fig. 2d and Supplementary Table 3).

### Ratio-based reference datasets

**Fig. 3:**
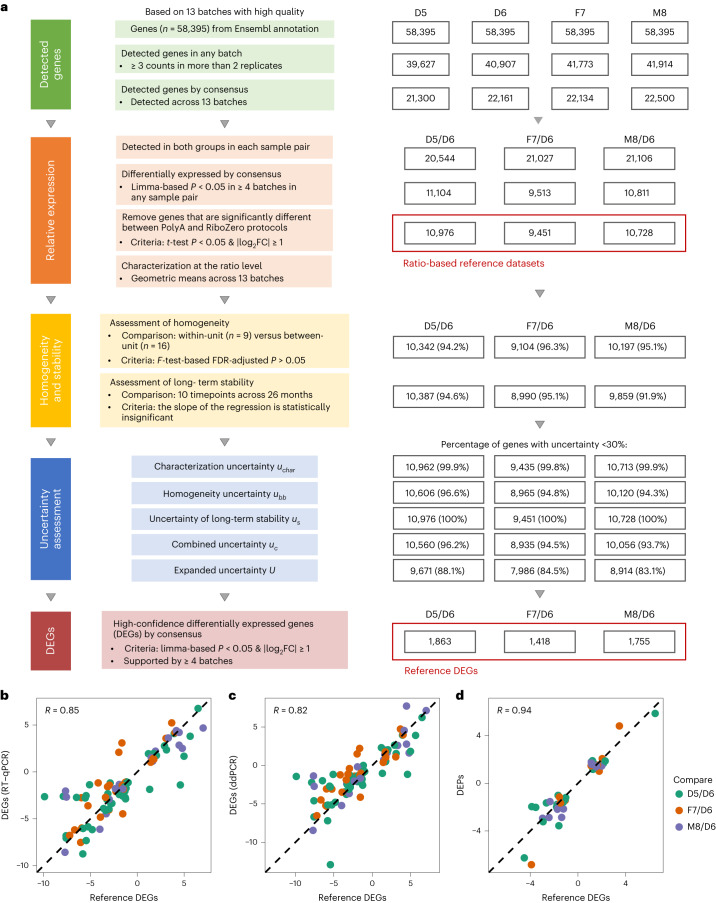
Construction and validation of ratio-based transcriptome-wide reference datasets. **a**, Workflow for constructing Quartet RNA reference datasets. Reference datasets were constructed according to the following steps: (1) identifying detectable genes; (2) calculating ratio-based expression based on reliably detectable genes that were differentially expressed; (3) assessing the homogeneity and stability of RNA reference materials; (4) assessing the uncertainty of ratio-based reference datasets; and (5) identifying high-confidence DEGs. **b**–**d**, Scatter plots of log_2_ fold changes (FCs) of gene expression between reference DEGs and RT–qPCR (**b**), ddPCR (**c**) and proteomics data (**d**). Pearson correlation coefficient across three sample pairs was calculated. Genes/proteins that were considered as differentially expressed in both methods shown in *x* axis and *y* axis were used for plotting. *x* axis: average log_2_FC from 13 high-quality RNA-seq batches reference DEGs. *y* axis: for the RT–qPCR and proteomics data, a gene or a protein was considered as a DEG/DEP when the *t*-test two-sided *P* < 0.05 and FC ≥2 or ≤0.5; for ddPCR data, genes that were identified as DEGs based on RT–qPCR were used. Average log_2_FC of RT–qPCR (*n* = 3), ddPCR (*n* = 2) and proteomics (*n* = 3) data from DEGs/DEPs were used for plotting. DEP, differentially expressed protein.

We next constructed transcriptome-wide reference datasets based on multi-batch and high-quality RNA-seq datasets, providing ‘ground truth’ for benchmarking. Ratio-based expression profiles, defined as a ratio or a fold change of expression levels between two sample groups for the same gene, agreed well across multiple transcriptomic technologies, including RNA-seq, microarray and quantitative polymerase chain reaction (qPCR)^15,20^. On the other hand, the incomparability of conventional ‘absolute’ expression profiles across different batches prevented meaningful cross-batch data integration^15,20^. Hence, we constructed the ratio-based transcriptome-wide reference datasets (Fig. 3a).

First, the detectable genes in each sample group (D5, D6, F7 or M8) were identified by consensus separately. In brief, if a gene was detected in all 13 batches in a sample group, it was considered expressed in that sample group. For the four Quartet reference materials (D5, D6, F7 and M8), 21,300, 22,161, 22,134 and 22,500 genes were expressed, respectively, representing 36.5–38.5% of the 58,395 genes annotated in GRCh38 (Fig. 3a). Moreover, around 32,104–33,937 genes (55–58%) were detected in more than four high-quality batches.

Second, ratio-based expressions (as log_2_ transformed) were calculated for three pairs of sample groups using replicates of D6 as the common denominator (D5/D6, F7/D6 and M8/D6). To improve the reliability of the reference values, genes that were satisfied with thresholds of *P* < 0.05 in each sample pair were used. Furthermore, genes that were significantly different (*P* < 0.05 and fold change ≥2 or ≤0.5) between PolyA and RiboZero protocols were removed to minimize technical variations introduced by the differences between the two distinct library preparation protocols. After these filtrations, the number of retained genes was 10,976, 9,451 and 10,728 for the three sample pairs (D5/D6, F7/D6 and M8/D6), respectively (Fig. 3a). Ratio-based reference datasets were then characterized between each pair of samples for a gene and were provided in the format of a geometric mean by summarizing from the 13 ratios calculated from each of the 13 high-quality RNA-seq datasets (Supplementary Tables 4 and 5).

Third, the homogeneity and stability of the Quartet RNA reference materials were assessed (Fig. 3a and Supplementary Fig. 9). Homogeneity and stability are two crucial characteristics of reference materials^37^. Homogeneity assessment aims to ensure that the previously characterized properties of reference materials are uniformly distributed across packaging units of the reference materials. Because the Quartet RNA reference materials were characterized using gene expressions, homogeneity assessment was conducted based on gene expression data. Here, we evaluated the homogeneity of reference materials by calculating within-unit (*n* = 9) versus between-unit (*n* = 16) variances of each gene using the analysis of variance (ANOVA) method (Supplementary Fig. 9a,b). Most (94.2–96.3%) genes performed well in homogeneity assessment (Supplementary Table 6). On the other hand, stability assessment aims to ensure that the value of the properties of the reference materials previously characterized remains unchanged over time. Here, we evaluated the stability of the Quartet RNA reference materials by calculating the slope of the regression of each gene based on the 15 batches of RNA-seq datasets that were generated from 10 timepoints over 26 months (Supplementary Fig. 9c,d). Most genes (91.9–95.1%) performed well in long-term stability assessment (Supplementary Table 6). Therefore, the Quartet RNA reference materials stored at −80 °C were homogenous and stable, as can be seen from the corresponding reference datasets.

Fourth, uncertainties of the reference materials were estimated. It is essential for identifying each source of uncertainties and to quantify the uncertainty introduced by each source. According to ISO Guide 35 (2017)^37^, ISO/IEC Guide 93-3 (2008)^38^ and SAC JJF-1343 (2012)^39^, the source of uncertainties can be classified into characterization uncertainties (*u*_*char*_), sample inhomogeneities (between-bottle variation, *u*_*bb*_) and instabilities (*u*_*s*_). These values were then aggregated to form the combined uncertainties (*u*_*c*_) and expanded uncertainties (*U*) with an expansion factor (*k* = 2, 95% confidence level) (Fig. 3a and Supplementary Table 6). As a result, most genes (83.1–88.1%) showed limited expanded uncertainties of less than 30%, demonstrating that the characterization of reference datasets was valid.

Finally, high-confidence DEGs in the reference datasets (reference DEGs) were identified. A gene was considered as a reference DEG between two sample groups if it was concordantly discovered as an upregulated or downregulated gene (*P* < 0.05 and fold change ≥2 or ≤0.5) in more than four of the 13 high-quality batches. The number of reference DEGs was 1,863, 1,418 and 1,755 for the D5/D6, F7/D6 and M8/D6 sample pairs, respectively (Fig. 3a and Supplementary Table 7).

To verify the reliability of the reference datasets, we performed qPCR with reverse transcription (RT–qPCR) as an orthogonal validation. We selected 82 genes from the Quartet RNA reference datasets and conducted RT–qPCR experiments on the four RNA reference materials (Supplementary Table 8). There is a high level of concordance between the Quartet reference datasets and the RT–qPCR data in terms of DEGs (92%, 91 of 99 DEGs across three sample pairs). We also compared the fold change of RT–qPCR versus that of reference datasets for the DEGs that were detected by both technologies (*n* = 91) (Supplementary Table 9). We observed an expected high level of concordance to RT–qPCR (*R* = 0.85), similar to what was previously reported between microarray and RT–qPCR (*R* = 0.80–1)^13^ (Fig. 3b). DEGs that were identified in the reference datasets and RT–qPCR were further validated using droplet digital PCR (ddPCR). Similar results were observed when comparing the fold changes between ddPCR and reference datasets in the aforementioned DEGs (Fig. 3c and Supplementary Table 9). Note that the level of the correlation coefficients depends on the level of the intrinsic biological differences between sample pairs under comparison. The differences among the Quartet RNA reference materials were relatively small compared to those of the MAQC samples A and B, resulting in relatively lower concordance^20^ between the reference datasets and the RT–qPCR or ddPCR data for the Quartet reference materials.

Moreover, we used a liquid chromatography with tandem mass spectrometry (LC–MS/MS)-based proteomics dataset (batch code: NVG_QEHFX)^32,40^ for cross-omics validation of the RNA reference datasets (Supplementary Table 10). When all detected genes and proteins were considered, the correlation between RNA-seq and proteomics was modest (*R* = 0.45–0.57) (Supplementary Fig. 10), which was similar to what was reported in previous studies (*R* = 0.36–0.60)^41,42^. However, we found that, for DEGs, there was a much higher concordance between RNA and protein data. When using DEGs that were detected by both RNA and protein measurements, the correlation increased to 0.94–0.96 (Fig. 3d and Supplementary Fig. 10). Thus, the protein-coding genes in the reference datasets that were differently expressed in the three sample pairs were successfully validated by the corresponding differential protein abundances. In addition, our findings indicated that the RNA reference datasets might also help benchmark proteomics technologies.

### Reference-dependent quality metrics

To benchmark RNA-seq data based on the aforementioned reference datasets, we developed three reference-dependent quality metrics. Specifically, we introduced the ‘relative correlation’ (RC) metric (that is, the Pearson correlation coefficient between the ratios of a test dataset for a given pair of samples and the corresponding ratio-based reference datasets, representing the trend of numerical consistency of the ratio-based expression profiles). We then introduced the ‘RMSE’ metric (that is, root mean square error (RMSE) of differences of ratios between a test dataset for a given pair of samples and the corresponding ratio-based reference datasets, representing the magnitude of average distances of ratio-based expression profiles). Moreover, we introduced the ‘MCC of DEGs’ (MCC) metric (that is, Matthews correlation coefficient (MCC) to measure the consistency of DEGs detected from a test dataset for a given pair of samples with those from the high-confidence DEGs in the reference datasets). Based on their definitions, higher values of RC and MCC of DEGs indicate a better fit between the test dataset and the reference dataset, whereas lower values of RMSE indicate a better fit. All three metrics were able to clearly demonstrate differences in data quality among the 21 batches of data, including the 13 high-quality and eight low-quality batches of data (Supplementary Fig. 11a).

One might argue that the lower RC, higher RMSE or lower MCC values of the eight pre-defined low-quality batches might have resulted from their exclusion during the construction of the reference datasets. To determine whether it was the case or not, we performed a 30-times cross-validation test. In brief, in each round, we randomly selected 13 batches from the 21 batches to ‘train’ the reference datasets. Reference-dependent quality metrics were then calculated, and the remaining eight batches were used as a ‘validation’ set. The results showed that the ‘train’ and ‘validation’ metrics were highly correlated (*R* = 1) (Supplementary Fig. 11b), demonstrating that the quality metrics were not dependent on whether the batches were included in the construction of the reference datasets or not. Instead, the three metrics objectively reflected the intrinsic quality of a dataset, indicating that they were suitable for performance evaluation of future datasets. The cutoff values of RC, RMSE and MCC values were set to 0.89, 0.38 and 0.54, respectively, which were expressed as the (mean − s.d.) of RC and MCC and the (mean + s.d.) of RMSE across validation sets in the 30-times cross-validation analysis (Supplementary Fig. 11b and Supplementary Table 3).

Furthermore, we compared characteristics between the two categories of quality metrics, including reference-independent quality metric (SNR) and reference-dependent quality metrics (RC, RMSE and MCC). In most cases, high-quality batches showed higher values of SNR, RC and MCC and lower values of RMSE, and vice versa, except for one batch (L5_B3) (Supplementary Fig. 11a). In this batch, a high SNR value (16.1) with low reference-dependent quality metrics (RC = 0.784, RMSE = 0.735 and MCC = 0.480) was observed. In fact, a customized library preparation kit designed for removing several highly expressed RNAs (for example, *RN7S* genes) was used in this batch (L5_B3), leading to overall differences between expression profiles from this batch and the reference datasets. Moreover, the complementarity between reference-independent and reference-dependent quality metrics was observed, indicating that both categories of quality metrics should be included in comprehensive performance assessment.

Finally, we calculated a total quality score by summarizing the two categories of quality metrics. Considering the high correlation among the three reference-dependent metrics (RC, MCC and RMSE) (absolute *R* ≥ 0.92) (Supplementary Fig. 11a), we used RC to represent the reference-dependent metric score for calculating the total quality score. The total quality score was expressed as the geometrical mean of SNR and RC for measuring the overall quality of a dataset for the intra-batch proficiency.

### Ratio-based expressions improve cross-batch reproducibility

In large-scale projects, expression profiles are usually measured across multiple batches and pooled together for downstream analysis. Cross-batch reproducibility is, therefore, crucial. Multi-batch RNA-seq datasets derived from the Quartet RNA reference materials allowed us for objective performance assessment of cross-batch reproducibility at multiple levels, including cross-time, cross-laboratory, cross-platform and cross-protocol.

**Fig. 4:**
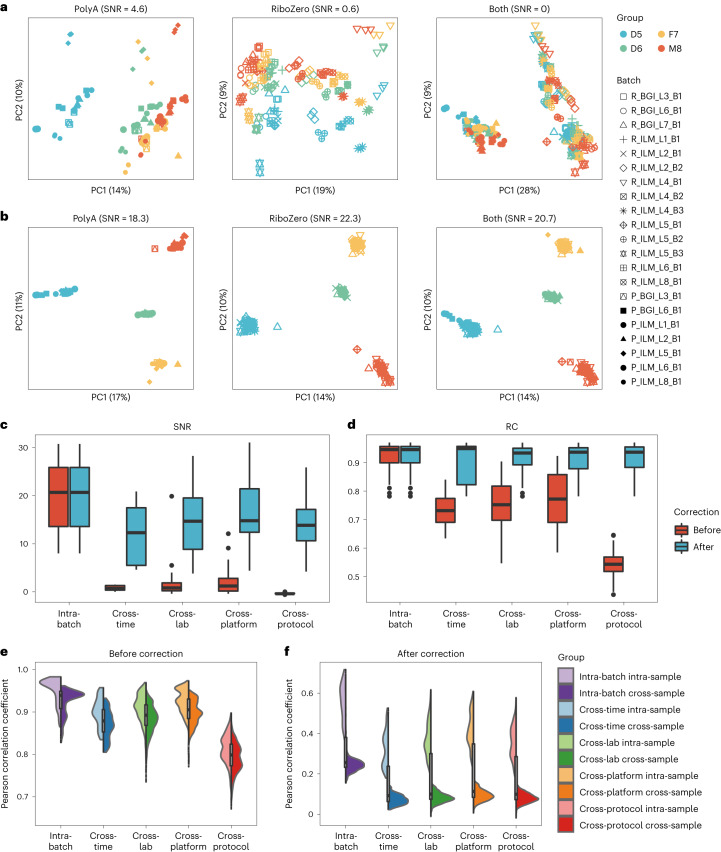
Performance evaluation of cross-batch reproducibility. **a**,**b**, Scatter plots of PCA on RNA-seq data before batch correction (**a**) and after correction (**b**) from replicates of the Quartet RNA reference materials (marked in colors) in the 21 batches (marked in shapes). Expressions in log_2_FPKM were used as before batch-correction datasets. Ratio-based expressions (which referred to converting expression profiles to gene-wise relative-scale profiles within each batch) were used to correct batch effects. Ratio-based expressions were obtained by subtracting log_2_FPKM by the mean of log_2_FPKM of the three replicates of D6 in the same batch. We used a multi-batch RNA-seq dataset, including 168 RNA-seq libraries from the RiboZero protocol and 84 RNA-seq libraries from the PolyA protocol. Plots were color-coded by sample groups and shaped by batches. **c**,**d**, Box plots of SNR values (**c**) and relative correlation with reference datasets (RC) values (**d**) for comparisons indicated at the *x* axis. When each batch of libraries was compared against each other, they could be classified into five different scenarios with increasing degree of differences, including intra-batch, cross-time, cross-laboratory, cross-platform of sequencing and cross-protocol levels. Intra-batch SNR values were calculated using 12 samples in the same batch, whereas SNR values of cross-batch were calculated by combining expression data from all combinations of two batches (*n* = 24). **e**,**f**, Violin plots of Pearson correlation coefficients based on expression profiles before (**e**) and after (**f**) batch correction for comparisons indicated at the *x* axis. D5, F7 and M8 samples were used to calculate pairwise correlations, whereas D6 samples were used as denominators for calculating ratio-based expressions for correcting batch effects. The number of combinations (*n*) used to derive statistics in **c**–**f** in each box were as follows: **c**: intra-batch, *n* = 21; cross-time, *n* = 7; cross-laboratory, *n* = 62; cross-platform, *n* = 43; cross-protocol, *n* = 98; **d**: intra-batch, *n* = 63; cross-time, *n* = 21; cross-laboratory, n = 186; cross-platform, *n* = 129; cross-protocol, *n* = 294; **e** and **f**: intra-batch intra-sample, *n* = 189; intra-batch cross-sample, *n* = 567; cross-time intra-sample, *n* = 189; cross-time cross-sample, *n* = 378; cross-laboratory intra-sample, *n* = 1,674; cross-laboratory cross-sample, *n* = 3,348; cross-protocol intra-sample, *n* = 1,161; cross-protocol cross-sample, *n* = 2,322; cross-platform intra-sample, *n* = 2,646; cross-platform cross-sample, *n* = 5,292.

In this study, after pooling batches of data from the PolyA and/or RiboZero protocol(s) together without batch corrections, the impact of batch effects on obscuring the differentiation of biologically distinct groups could be clearly seen in a PCA plot with a diminished SNR value of below 5 (0–4.6) (Fig. 4a). Non-experimental factors, rather than intrinsic biological groups (D5, D6, F7 and M8), exhibited the largest differences. When PCA was based solely on the MAQC samples without the Quartet samples, batch effects could not be observed from PC1 due to the overwhelming biological differences between the two MAQC samples. However, indications of batch effects were appreciable from PC2 (Supplementary Fig. 12). When ratio-based expressions were used, which referred to converting expression profiles to gene-wise relative scale within each batch using D6 as the denominator, the SNR value increased to around 20 (18.3–22.3). Meanwhile, all libraries from the PolyA and RiboZero protocols of the same sample group were grouped together based on ratio-based expressions (Fig. 4b). Similar results were observed when an alternative gene quantification tool (for example, RSEM) or a normalization method (for example, normalized counts) was used to quantify and compare relative expressions (Supplementary Figs. 13 and 14). These findings indicate the critical importance of detecting and correcting batch effects in multi-batch studies. Notably, ratio-based expressions were effective in mitigating such batch effects.

We then compared pairwise cross-batch performance to investigate integrability at different levels. When different batches of libraries are compared against each other, they could be classified into five different scenarios with increasing degree of differences, including intra-batch, cross-time, cross-laboratory, cross-platform of sequencing and cross-protocol levels. We compared the consistency between datasets from different levels of comparison using three quality metrics: SNR, RC and Pearson correlation coefficients.

SNR values were calculated for the five scenarios of comparisons. Compared to intra-batch SNR values (median SNR = 20.7), SNR values dropped to −0.4–1.2 (median SNR) at cross-time, cross-laboratory, cross-platform or cross-protocol comparisons when absolute expressions (log_2_FPKM) of the two datasets were merged to calculate the SNR value. In this case, it is essentially impossible to distinguish different sample types under the influence of ‘batch effects’. Thus, expression profiles from two batches of libraries could not be integrated directly at the absolute expression level. However, when ratio-based expressions were used, SNR values maintained as high as 12.3–14.8 (median SNR) (Fig. 4c). This finding again reinforced the previous notion that ratio-based expressions are much more resistant to batch effects (Fig. 4b).

Similar results were obtained for performance based on RC values. Compared to intra-batch RC (median RC: 0.946), RC values dropped to 0.543–0.772 (median RC) when absolute expressions of two datasets were compared. However, they maintained at 0.933–0.949 (median RC) when ratio-based expressions of the two datasets were considered (Fig. 4d).

Additionally, the median correlation of absolute expressions was as high as 0.965 for intra-batch technical replicates and 0.938 between different groups in the same batch. It dropped to 0.814–0.927 for cross-batch technical replicates. What is worse, correlations of technical replicates for the same sample from difference batches were significantly lower than correlations between different sample groups from the same batch (*P* < 0.001), highlighting the critical impact of batch effects (Fig. 4e). On the contrary, correlations of ratio-based expressions of technical replicates (0.319 –0.401) were consistently higher than those of different groups (0.072–0.093) under the different levels of cross-batch comparisons (Fig. 4f), demonstrating the differentiating power at the ratio-based expression level.

Our findings support the important roles of reference materials in assessing cross-batch reproducibility and their effectiveness in removing batch effects. It should be noted that we could clearly observe/monitor batch effects based on multi-batch datasets of Quartet RNA reference materials (Fig. 4a,b), whereas it is impossible with the MAQC reference materials due to their substantial differences (Fig. 1b). Thus, the Quartet reference materials can provide more precise assessment of measurement performance based on their small but biologically relevant intrinsic differences, highlighting their critical roles in assessing cross-batch reproducibility.

### Biological differences between the Quartet twins

**Fig. 5:**
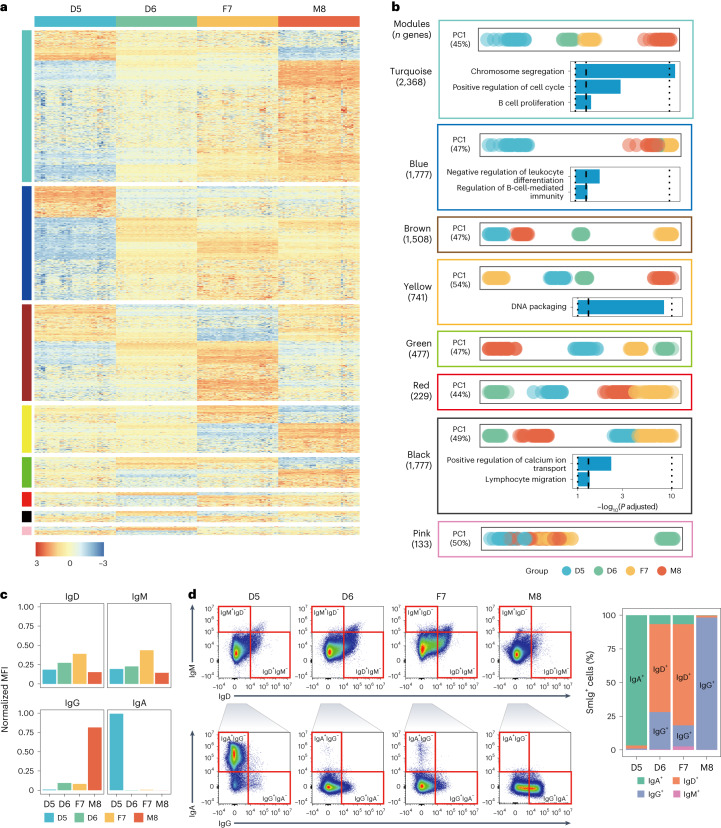
Biological differences between immortalized B-LCLs of the Quartet monozygotic twins. **a**, Expression profiles from co-expression modules using data from 13 batches with high quality. Color-coded module membership was displayed in the color bars to the left of the dendrograms. Ratio-based expressions were obtained by subtracting log_2_FPKM by the mean of log_2_FPKM of the three replicates of D6 in the same batch. The heat map was colored using *z*-scored ratio-based expression profiles. **b**, Distances of samples in PC1 space and list of GO terms enriched with genes in each corresponding module. Enriched GO terms were generated based on hypergeometric test using clusterProfiler^64^, with a Benjamini–Hochberg correction and an adjusted *P* value cutoff of 0.05. PC plots were colored by sample groups. Bar plots were colored based on the number of genes included in GO terms. **c**, The normalized expression level (median fluorescence intensity, MFI) of B cell surface membrane SmIg IgD, IgM, IgG and IgA in immortalized B-LCLs. **d**, Left: representative flow cytometric dot plots show the IgD^+^ cells, IgM^+^ cells, IgG^+^ cells and IgA^+^ cells in immortalized B-LCLs. Right: expression levels of IgA, IgG, IgM or IgD in the four immortalized cell lines.

It was noticed that the two LCLs corresponding to the two monozygotic twin daughters (D5 and D6) exhibited consistently large differences in gene expression in all batches of data (Supplementary Figs. 6 and 15), although one might have expected that the expression profiles from the two identical twins would show the highest similarity among all six pairs of the Quartet sample groups. Here, we used ratio-based expression profiles of the 13 high-quality batches and applied a weighted gene co-expression network analysis (WGCNA) approach^43^ to discern the underlying biological forces behind the differences in transcriptome between the two cell lines. Genes were grouped with strong co-expression patterns across the sample set into eight modules (Fig. 5a). D5 samples were distinct from D6 samples in the PC1 space based on transcriptomic expression for most modules (seven of eight modules), including the largest module (turquoise module) with 2,368 highly co-expressed genes (Fig. 5a,b). Functional analysis showed that the turquoise module genes were enriched in Gene Ontology (GO) terms, such as cell cycle and B cell proliferation (Fig. 5b). Moreover, a 1,777-gene module (blue module), which showed dispersity between D5 and other three groups (D6, F7 and M8) in the PC1 space, was enriched in B-cell-mediated immunity. These results imply that differential processes of B cell subtype selection and effects of cell culture might have occurred among the Quartet RNA reference materials (Fig. 5b).

On the contrary, when we applied WGCNA analysis on the log_2_FPKM values (Supplementary Fig. 16), the two largest modules (turquoise and blue) were grouped according to protocols and/or batches. Only for the third (brown) and fourth (yellow) largest modules, the samples were grouped based on donors with genes enriched in B cell activation and immune responses. These results imply that biological signals of relationships among the Quartet cell lines were largely masked at the raw FPKM level, highlighting the negative impact of batch effect in absolute profile data.

To identify B cell subtypes corresponding to the Quartet RNA reference materials, we examined the expression levels of B cell surface membrane immunoglobulins (SmIg) on the Quartet cell lines. Four types of SmIg were measured, including IgD, IgM, IgG and IgA, which were biomarkers of the developmental stages of B cells. Notably, the IgA expression pattern of the immortalized cell lines from the two monozygotic twin daughters (D5 and D6) exhibited substantial differences in that IgA was highly expressed in D5 but almost undetectable in D6 (Fig. 5c). Additionally, the expression level of IgG was much higher in the M8 group compared to the other three groups (Fig. 5c). We further performed immunophenotypic analysis of the four immortalized cell lines. In agreement with the SmIg findings from RNA-seq, the IgA^+^ cells were mainly present in the cell line from D5, whereas a lower percentage of IgA^+^ cells was found in other cell lines (Fig. 5d and Supplementary Fig. 17). Furthermore, the percentage of IgG^+^ cells was higher in M8 compared to the other three groups (Fig. 5d).

We hypothesized that the major factors driving transcriptomic expression characteristics were probably related to the processes for immortalizing cell lines (for example, B cell subtype selection during EBV infection and cell culture)^44^. To validate this hypothesis, we further conducted RNA-seq experiments based on whole-blood samples of the four donors. Expression profiles of whole-blood samples from D6 and F7 donors looked different (Supplementary Fig. 18a) and were not grouped together as what we observed based on expression profiles from the cell lines (Supplementary Fig. 15). On the other hand, the twin daughters grouped close to each other and showed the highest similarity in expression profiles among the Quartet samples in the PCA plot (Supplementary Fig. 18b). The intrinsic biological differences between the Quartet monozygotic twins enhanced our understanding of the Quartet RNA reference materials and could be used as another layer of built-in truth to increase the quality control utilities of the Quartet RNA reference materials^45^.

### Recommended group–replicate combinations

**Fig. 6:**
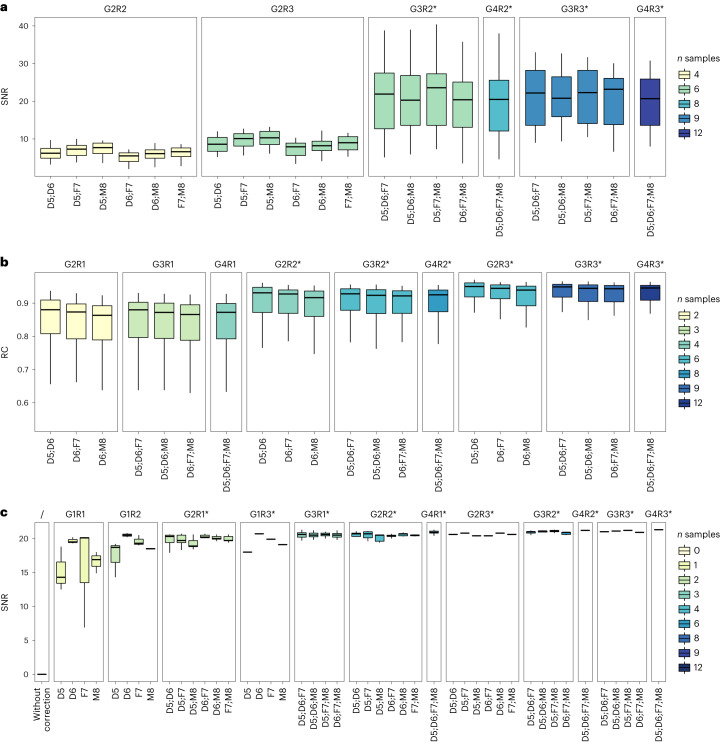
Recommended group–replicate combinations for using the Quartet RNA reference materials for quality control. **a**,**b**, Distribution of SNR (**a**) and relative correlations with reference datasets (RC) (**b**) under different group–replicate combinations of Quartet RNA reference materials used for assessing intra-batch proficiency. **c**, Distribution of SNR values for ratio-based expression using different numbers of samples and/or replicates as the denominator for the calculation of the ratio-based expressions. *x* axis represents the enumerated number and groups of Quartet reference materials. Titles of subpanel represent the number of sample groups (G) and replicates (R) used for calculating SNR (**a**), RC (**b**) and denominators for applying the ratio-based method (**c**). For example, ‘G2R2’ represents four libraries comprising two sample groups (G) with two replicates (R) per group. The recommended combinations are marked with asterisks (*).

An important question is what group–replicate combinations would constitute an appropriate choice for applying Quartet RNA reference materials for quality control in routine transcriptomic profiling. Thus, the replicate number and group number of Quartet RNA reference materials that could be used were enumerated. The results revealed that a minimum of three sample groups and two replicates per batch were required for reaching SNR with high sensitivity for distinguishing data quality of different batches. The use of only two sample groups was not enough for distinguishing quality difference of datasets (Fig. 6a). Meanwhile, it was revealed that a minimum of two replicates per sample type were required for obtaining RC with high consistency with the ground truth (Fig. 6b). Under the same number of replicates and groups, the impact of group combinations (D5, D6, F7 or M8) was minor.

Furthermore, the number and groups of reference materials that could be used as a reference (denominator) in ratio-based profiling within each batch were enumerated. SNR increased markedly at the ratio (relative) level compared to the absolute level even when only one single replicate was used, with a median SNR value greater than 14 (Fig. 6c), and the SNR values further increased when more replicates and/or more sample groups were added to calculate average expression values as the denominator. Moreover, the SNR values obtained using only one sample to calculate the denominator varied greatly, whereas SNR values based on the mean of more replicates and/or sample groups as the denominator were more stable. Given the same number of samples as the denominator, a higher number of sample groups helped further increase SNR.

These results provided a solid foundation to determine the optimal number of samples and/or replicates to be used for performance assessment and ratio-based transcriptomic profiling using the Quartet RNA reference materials. When RC with the reference datasets was used for intra-batch performance assessment (Fig. 6b) and when multiple-sample groups/replicates were used as the denominator for ratio-based cross-batch effect correction (Fig. 6c), the use of two sample groups appears sufficient in many settings. However, when reference-dataset-free SNR was used for intra-batch performance assessment (Fig. 6a), multiple groups of reference materials (≥3) are required. SNR has been shown to be more sensitive in assessing and diagnosing data quality issues. Moreover, SNR is a reference-independent quality metric, enabling assessment beyond the boundaries of the reference datasets. Hence, for proficiency test purposes, multi-group references are needed to implement comprehensive quality assessment.

## Discussion

We generated well-characterized, high-quality, homogenous and stable Quartet RNA reference materials and constructed corresponding reference datasets from reliable transcriptomic data, which can be a useful tool for objectively assessing data quality and improving the reliability of transcriptomic profiling, specifically within a clinical setting. Notably, the Quartet RNA reference materials have been approved by China’s State Administration for Market Regulation as the First Class of National Reference Materials and are extensively being used for proficiency testing and method validation.

The Quartet RNA reference materials exhibit several advantages. First, they are a part of multi-omics reference materials, with matched DNA, RNA, proteins and metabolites generated from the same immortalized cell lines. This study design allows for cross-omics validation and will help reliably understand the biological traits of the reference materials. Second, the suite of reference materials is from a four-member Quartet family including two monozygotic twin daughters and their father and mother. Genomic and phenotypic characteristics are involved in the four RNA samples, acting as built-in ‘truth’. The reference datasets based on intrinsic biological differences among the Quartet RNA reference materials have been constructed and can be used as ‘ground truth’ for quality assessment (Fig. 3). Expression characteristics affected by genetic relationships of the four reference materials will be further studied^45^. Third, the RNA reference materials are derived from cell lines from four individuals. The small intrinsic biological differences among the Quartet RNA reference materials enable precise assessment at inter-patient level, rather than inter-tissue level, which are closer to clinical scenarios of subtle differences among study groups (Fig. 1c). Fourth, the RNA samples are produced in large amounts in one batch and are renewable through cell culture. By minimizing batch effects that may be introduced during cell culture and RNA extraction, the Quartet RNA reference materials are sufficient for performing standard RNA-seq experiments over 10,000 to 50,000 times and provide a material basis for long-term quality monitoring. Based on comprehensive assessments, the Quartet RNA reference materials are homogenous and long-term stable at the storage temperature of −80 °C. The publicly available Quartet RNA reference materials can also be used for further evaluation of emerging technologies as well as new areas of interest that are beyond gene expression levels, such as AS, RNA editing, gene fusion and epitranscriptomics. In addition, the Quartet RNA reference materials comprise high-quality total RNAs, including not only full-length RNAs but also small RNA molecules such as miRNAs, enabling further quality assessment of small RNA profiling technologies.

Quality metrics derived from the Quartet RNA reference materials and reference datasets can be used for proficiency testing and external quality assessment. Previous quality metrics were focused on biases from library preparation or on detecting outliers in expression profiles^4,17,21,46–49^. It has been previously demonstrated that ‘lab effects’ strongly affect the detection of DEGs, highlighting the importance of assessing data quality in detecting DEGs^20^. In this study, we developed comprehensive quality metrics for assessing the reliability of differential expression, including discriminating power across different biological groups (SNR) and reproducibility of identifying DEGs (RC, RMSE and MCC), which are reference independent and reference dependent, respectively. In addition, distributions of these quality control measures were obtained from multiple real-world RNA-seq datasets, providing practical cutoffs to decide whether the proficiency of a test dataset is acceptable.

The Quartet RNA reference materials can be used for monitoring and correcting batch effects. Batch effects are notorious technical variations irrelevant to study factors and are challenging to deal with, especially when they are confounded with biological factors of interest^50,51^. Our results demonstrate that the presence of batch effects without correction can lead to misclassification of samples (Fig. 4a), but we found that these batch effects can be mitigated by using ratio-based expressions (Figs. 4b,c,f and 6c), if one or more common reference materials are profiled across batches. Our companion work found that using ratio-based data analysis by scaling the absolute feature values of study samples relative to those of concurrently measured reference sample(s) on a feature-by-feature basis could effectively mitigate the widespread problems of batch effects in epigenomics, transcriptomics, proteomics and metabolomics datasets^52,53^. This ratio-based method is equally effective even for study design of completely unbalanced distributions of samples in different groups between different batches. In practice, the imbalance in samples across batches is almost inevitable because of hidden biological subpopulation variabilities^50,51^.

In addition, the advantages of ratio-based expression profiles remain in identifying DEGs, which were extensively explored in our companion work^53^. A straightforward method (that is, fold change ranking with a non-stringent *t*-test *P* value cutoff) can be applied to perform differential analysis in ratio-based profiles. The effectiveness of the DEG method was previously shown in microarray^15,54^ and RNA-seq^18,20^ data in absolute expressions.

The Quartet RNA reference materials can act as valuable tools for quality control in large-scale, longitudinal and multi-center projects. Many large-scale consortium projects with comprehensive and coordinated efforts help accelerate understanding of the molecular basis of transcriptome by producing RNA-seq data with a large sample size^36,55–57^. However, the broad variety of platforms, protocols and laboratory proficiencies^58–60^ has created the need for comprehensive reference materials. At the starting point of a large-scale project, we recommend that researchers conducting RNA-seq experiments using the Quartet RNA reference materials in each laboratory assess and ensure intra-batch proficiency and cross-batch reproducibility before analyzing precious study samples. Meanwhile, researchers can use the Quartet RNA reference samples routinely along with study samples to monitor and correct batch effects.

The combinations of sample groups and number of replicates for the application of the Quartet RNA reference materials are context dependent. For proficiency test and external quality assessment purposes, where the frequency of reference usage could be as low as a few times per year, it is recommended to apply multiple groups of samples with multiple replicates per group. Users can apply a minimum of three sample groups and two replicates for quality assessment (Fig. 6). Users can apply a total of 12 samples, comprising the four Quartet RNA reference materials with three replicates for each RNA sample group, to implement full quality assessment mentioned in the study and remove batch effect in a robust way (Fig. 6a,b). For batch effect removal purposes in large cohort studies, where the Quartet RNA reference materials are expected to be routinely used along with study samples, and where additional cost associated with profiling reference samples becomes an issue, it is recommended to apply fewer sample groups and fewer replicates per batch. Users can even apply four sample groups or as few as two sample groups without replicates as a cost-effective choice of references for monitoring and correcting batch effects (Fig. 6c). In this case, we suggest the use of a total of four profiles from each replicate of the four Quartet RNA reference materials as the denominator per batch of 96 libraries for ratio-based expression profiling, reaching a high SNR while maintaining a reasonable additional cost (4 / (96 − 4) = 4.3%) per batch of 92 study samples.

To facilitate the adoption of multi-omics reference materials, reference datasets and quality metrics from the Quartet Project, we developed a Quartet Data Portal (http://chinese-quartet.org/) for access to the Quartet resources and for enhancing the quality consciousness of the community^40^. Researchers can request the multi-omics reference materials, datasets and reference datasets from the data portal. Additionally, researchers can upload RNA-seq data of their own, automatically analyze and evaluate data quality and/or share data with the community. With the growing use of the Quartet reference materials, we hope to generate and collect diverse datasets and further upgrade the reference datasets.

Although many advantages of using the Quartet RNA reference materials were obvious and are listed above, several limitations of the Quartet samples should also be noted. First, only around 55–58% of the 58,395 annotated genes were reliably detected (detected in more than four high-quality batches) in the Quartet RNA reference materials, limiting quality assessment and ratio-based scaling to these detectable genes. This is not a serious issue when using Quartet RNA reference materials for proficiency testing and external quality assessment. However, this could become a limitation when the Quartet RNA reference materials are to be used for profiling along with study samples for reporting ratio-based profiling data. Ratio-based scaling may successfully mitigate batch effects from genes when they are expressed in both the study samples and reference material(s). If a gene is expressed only in study samples but not in reference materials, its expression may not be successfully corrected. In such a scenario, a fudge factor may be used for making the ratio calculation possible. The limitations of ratio-based scaling are also extensively discussed in our accompanying papers^52,53^. Second, a single analysis pipeline was used in this study, which may introduce bias in transcriptomic quantification and characterization of the reference datasets. Although previous studies compared the performance of different RNA-seq analysis tools and found overall good reproducibility for different tool combinations in terms of differential expression calls after proper filtering processes^61–63^, bioinformatics tools will be further evaluated and used for characterizing the reference datasets. Third, the datasets were generated by high-throughput short-read sequencing technologies. It is likely that, with further benchmarking and widespread adoption of reference materials, additional reagents, protocols and instruments will be evaluated.

In summary, the Quartet RNA reference materials and reference datasets are unique resources to improve quality of RNA-seq data. Inclusion of the Quartet RNA reference materials in RNA-seq batches coupled with reference datasets will make RNA-seq more reproducible, accurate and comparable, especially within clinical settings.

## Methods

### Cell lines

Human subjects, establishment of the EBV-transformed B-LCLs, expansion and cryopreservation of the cells, cell culture and cell quality control are described in an accompanying paper by Zheng et al.^52^. In brief, four healthy volunteers from a quartet family in Taizhou, Jiangsu, China were enrolled, and their peripheral blood samples were collected. The study was approved by the institutional review board of the School of Life Sciences, Fudan University (BE2050). Peripheral blood mononuclear cells were isolated; the naive B cells were sorted and infected with EBV by centrifugation at 400*g* for 1 h; and the immortalized cell lines were cultured in an incubator. About 1.0 × 10^11^ cells were harvested for each cell line in the same batch to ensure that multi-omics reference materials were extracted from the same batch of cultured cells. About 2.0 × 10^9^ cells per cell line were used for generating Quartet RNA reference materials.

### RNA extraction and quality assessment

TRIzol reagent was added to resuspend the cells. Total RNA was extracted using an RNeasy Maxi Kit (Qiagen, cat. no. 75162) including on-column DNase-I digest, according to the manufacturer’s instructions.

RIN values were obtained for assessing RNA quality with a Bioanalyzer 2100 (Agilent Technologies) using RNA 6000 Nano assay (Agilent Technologies) and a Qsep 100 system (BiOptic). RNA concentrations, OD280/260, OD260/230 and 28/18S were assessed by a NanoDrop ND-2000 spectrophotometer (Thermo Fisher Scientific). Over 5 mg of RNA was obtained per cell line. RNAs were then aliquoted into more than 1,000 tubes per sample group with 5 μg of RNA per tube.

As a part of the Quartet Project, multi-omics reference materials (DNA, RNA, protein and metabolite) were established simultaneously from the same batch of cultured EBV-immortalized B-LCLs from the Quartet family members. The Quartet multi-omics reference materials are available to the public. Users can request reference materials via the Quartet Data Portal (http://chinese-quartet.org/).

### RNA stability assessment

#### Bottle-opening and freeze–thaw stability

RNAs were stored in 0.5-ml tubes at −80 °C for over 1 h until completely frozen. Frozen samples were thawed at 4 °C for approximately 0.5 h until completely thawed (freeze–thaw 1). We then opened the tubes and took 1-μl aliquots per tube out for further analysis (bottle-opening 1). The remaining RNAs were immediately re-frozen at −80 °C. This cycle was repeated for 20 times. RIN values were assessed at the 0, 1, 2, 3, 4, 5, 6, 8, 10, 14, 16, 18 and 20 times of opening and freeze–thaw to evaluate the integrity of RNA. Three replicates per sample group were assessed during each assessment.

#### Short-term stability

The stability of Quartet RNA reference materials at room temperature (22–25 °C) and 4 °C was assessed. First, four groups of the Quartet RNA reference materials were assessed for up to 4 d. RIN values were assessed at 0 h, 6 h, 24 h and 4 d to evaluate the overall quality of RNA during storage. Second, considering the same trends and similar results across the four Quartet RNA reference materials, we used two RNA reference materials (F7 and M8) for up to 14 d. RIN values were assessed at 0, 2, 4, 5, 6, 7, 8, 10, 12 and 14 d, separately. Three replicates per sample group were assessed during each assessment.

#### Long-term stability

The stability of RNA reference materials at storage of −80 °C was monitored for up to 20 months. RIN values were assessed at 0, 1, 2, 3, 4, 5, 6, 7, 8, 9, 11, 12, 13, 15, 16, 17 and 20 months. Three replicates per sample group were assessed at each timepoint. The MAQC RNA reference materials, including A sample (Universal Human Reference RNA, Agilent Technologies) and B sample (Human Brain Reference RNA, Thermo Fisher Scientific)^15^, were used as controls at each timepoint.

### Library construction and sequencing

According to the Quartet Project study design, 12 tubes of RNA samples were sent to each laboratory, including four groups of the Quartet RNA reference materials with triplicates per group. Library preparation, library quality control and sequencing were conducted in a fixed order (D5-1, D6-1, F7-1, M8-1, D5-2, D6-2, F7-2, M8-2, D5-3, D6-3, F7-3 and M8-3) in each laboratory to eliminate confounding factors, such as experimental sample processing order with sample group.

RNA-seq library preparation and high-throughput sequencing were conducted by each laboratory. In brief, libraries were constructed by PolyA selection or ribosomal RNA depletion (RiboZero) methods. The libraries were sequenced on Illumina NovaSeq (ILM) or MGI DNBSEQ-T7 (BGI) platforms with paired-end (PE) reads of 100–150 base pairs (bp). A total of 252 Quartet RNA-seq libraries from 21 batches were generated. Additionally, we simultaneously generated 20 batches of RNA-seq datasets using MAQC reference materials as controls. Detailed information on RNA-seq library construction and sequencing is shown in Supplementary Table 2.

Four RNA libraries from whole blood of the Quartet donors were constructed by the RiboZero method (TruSeq RNA Library Prep Kit) and sequenced on an Illumina HiSeq 4000 platform with 150-bp PE reads.

### Alignment and gene quantification

Preliminary processing of raw fastq reads was performed using fastp version 0.19.6 to remove adapter sequences^65^. Read alignment and quantification were conducted using HISAT version 2.1, SAMtools version 1.3.1, StringTie version 1.3.4 and Ballgown version 2.14.1 (ref. ^66^). Reference human genome build 38 (https://genome-idx.s3.amazonaws.com/hisat/grch38_snptran.tar.gz) and gene model from Ensembl (http://ftp.ensembl.org/pub/release-93/gtf/homo_sapiens/Homo_sapiens.GRCh38.93.gtf.gz) were used for read mapping and gene quantification. log_2_ transformation was then conducted based on FPKM values. To avoid infinite values, a value of 0.01 was added to the FPKM value of each gene before log_2_ transformation. Expression profiles based on detected genes were used for further analysis. A gene was considered detectable (expressed) in a biological group within a batch if ≥3 reads were mapped onto it in at least two of the three replicates. One replicate of MAQC B samples (library ID: R_ILM_L2_B1_B_3) was not included in further analysis due to low quality.

Moreover, we applied the RSEM tool for gene quantification to evaluate the impact of pipeline on assessing the reliability of RNA-seq data by comparing with the results from StringTie. Specifically, two batches of RNA-seq datasets from Quartet RNA reference materials were used, including one high-quality batch (R_ILM_L8_B1) and one low-quality batch (R_ILM_L4_B1) based on prior performance evaluation. Read alignment and quantification was conducted using Bowtie2 version 2.5.1 (ref. ^67^) and RSEM version 1.2.28 (ref. ^68^). Parameters were set by default according to the recommended pipeline from RSEM (https://github.com/deweylab/RSEM). The same reference genome and gene model were used. Expression matrix of FPKM values was obtained.

Quality control analysis of sequencing data at pre-alignment and post-alignment level was conducted using FastQC version 0.11.5 (ref. ^69^), FastQ Screen version 0.12.0 (ref. ^70^), Qualimap version 2.0.0 (ref. ^71^) and MultiQC version 1.8 (ref. ^72^).

### Validation of analysis pipeline based on MAQC reference materials

The bioinformatics pipeline was validated using published data from the MAQC RNA reference materials^15,20^. Specifically, we downloaded the published RNA-seq fastq files through the Gene Expression Omnibus (GEO) (GSE47774), analyzed the dataset using the bioinformatics pipeline used in this study and generated an expression matrix of FPKM values as the dataset for validating the reliability of our pipeline. Meanwhile, the expression matrix of count values of the same dataset was obtained from the R/Bioconductor package seqc version 1.28.0 (ref. ^20^), which could be used as positive control dataset. Data were normalized to counts per million (CPM). A value of 0.01 was added to the FPKM or CPM value of each gene, and log_2_ transformation was then conducted. Moreover, the expression profiles of the MAQC RNA reference materials from TaqMan assay were obtained through the GEO (GSE5350)^15^, which were used as the ‘ground truth’. Gene ID was mapped to Ensembl gene ID using the R/Bioconductor package biomaRt version 2.50.1. A total of 725 genes were mapped to Ensembl gene ID and were detected both in the RNA-seq and TaqMan data. Pearson and Spearman correlation coefficients based on log_2_ ratios (A/B) were further computed.

### Ratio-based expression

Ratio-based expression data were obtained within each batch on a gene-by-gene basis. Specifically, ratio-based expressions were calculated based on log_2_FPKM values. For each gene, the mean of expression profiles of replicates of reference sample(s) (for example, D6) was first calculated and then subtracted from the log_2_FPKM values of that gene in each study.

### DEGs

Differential expression analyses were implemented using the limma version 3.50.0 (ref. ^73^) and edgeR version 3.36.0 (ref. ^74^) packages according to guidelines from the limma package. A gene was considered differentially expressed in a batch between two sample groups if two-sided *P* < 0.05 and fold change ≥2 or ≤0.5 using the limma package for upregulation or downregulation, respectively.

### Identification and quantification of alternative splicing

The alignment results based on the HISAT2 were used to identify AS events using SplAdder toolkit^75^ with the default parameters. Six types of AS events were quantified using percent spliced in values, including exon skip, intron retention, alternative 3′ splice site, alternative 5′ splice site, cassette exon and coordinated mutually exclusive exons.

### Construction of reference datasets

We constructed the reference datasets of ratio-based expression based on the following steps: (1) identifying detectable genes; (2) calculating ratio-based expression based on reliable genes that were differentially expressed and with low uncertainty; (3) assessing the homogeneity and stability; (4) assessing the uncertainty of ratio-based reference datasets; and (5) calculating high-confidence DEGs in reference datasets.

First, detectable genes were identified. A gene was considered expressed in a sample in each batch if more than three reads were mapped to it in at least two of the three replicates. If a gene was detected in all the 13 batches in a sample group (D5, D6, F7 and M8), it was considered expressed in that sample group.

Second, ratio-based expressions were calculated. We used the expression profiles of three replicates of D6 in the same batch as the denominators and derived the ratio-based expressions for the three sample pairs (D5/D6, F7/D6 and M8/D6). The reference ratio-based expressions between each pair of samples for a gene were provided in the format of an average by summarizing from the 13 fold changes calculated from each of the 13 high-quality RNA-seq datasets. To improve the reliability of the reference values, genes were included if they satisfied the following criteria: (1) detectable across the two groups of each sample pair; (2) limma-based^73^ two-sided *P* < 0.05 in at least four batches in each sample pair; and (3) not significantly different between PolyA and RiboZero protocols (Student’s *t*-test two-sided *P* > 0.05 or fold change <2 and >0.5).

Third, the homogeneity and stability were assessed using RNA-seq datasets. The Quartet RNA reference materials were considered to be homogenous and stable, as can be seen from the corresponding reference datasets. Additionally, uncertainties of reference materials were assessed.

Finally, high-confidence DEGs in the reference datasets (reference DEGs) were identified. A gene was considered as a reference DEG between two sample types if it was concordantly discovered as an upregulated or downregulated gene (two-sided *P* < 0.05 and fold change ≥2 or ≤0.5) in more than six of the 13 high-quality batches.

### Homogeneity assessment based on RNA-seq datasets

The homogeneity of the Quartet RNA reference materials was assessed using RNA-seq data. We randomly selected 17 tubes (units) of each Quartet RNA reference material and named them as N1–N17. Under the same condition, nine replicates in the N1 tube and one replicate in tubes N2–N17 of each material were assessed to represent within-unit (*n* = 9) and between-unit (*n* = 16) characteristics. A total of 25 RNA-seq experiments per reference material were conducted.

RNA-seq libraries were constructed by ribosomal RNA depletion methods (VAHTS Universal V6 RNA-seq Library Prep Kit for Illumina) and sequenced on the Illumina NovaSeq platform with 150-bp PE reads. Alignment, quantification and quality control were conducted using the same analysis pipeline and parameters described above.

The within-unit and between-unit variances were then calculated using the ANOVA method^37,39^. Ratio-based expressions were obtained by subtracting log_2_FPKM by the mean of log_2_FPKM of the three replicates of D6 in the same batch and used. A gene was considered to be homogeneous when a cutoff of false discovery rate (FDR)-adjusted ANOVA-based two-sided *P* > 0.05 was used. Only between-unit homogeneity is studied, because within-unit homogeneity might be negligible in the case of intrinsically homogeneous materials, such as solutions^76^.

### Long-term stability assessment based on RNA-seq datasets

We assessed the long-term stability of the reference materials of 15 batches of RNA-seq datasets that were generated from up to 26 months. Ratio-based expressions were obtained by subtracting the mean log_2_FPKM of the three replicates of D6 in the same batch from the log_2_FPKM values. According to ISO Guide 35 (2017)^37^ and SAC JJF-1343 (2012)^39^, long-term stability assessment was conducted based on regression analysis. For each gene, the observed slope *b*_1_ and uncertainty of slope *b*_1_ ((*b*_1_)) was calculated. If |*b*_1_| < s(*s*(*b*_1_)) × *t*_0.95,*n*−2_, the expression of the gene is stable, and vice versa, where *t*_0.95,*n*−2_ is critical *t* value for a confidence level of 95% and *n* − 2 degrees of freedom.

### Uncertainty assessment of reference datasets

According to ISO Guide 35 (2017)^37^, ISO/IEC Guide 93-3 (2008)^38^ and SAC JJF-1343 (2012)^39^, the source of uncertainties can be classified into characterization uncertainties (*u*_*char*_), sample inhomogeneities (between-bottle variation, *u*_*bb*_) and instabilities (*u*_*s*_). These values were then combined to form the combined uncertainties (*u*_*c*_) with a simple additive measurement model using an equal weight of the three uncertainty sources, as recommended^37–39^. The expanded uncertainties (*U*) were further computed by multiplying *u*_*c*_ with an expansion factor.

First, characterization uncertainty of genes in the reference datasets was evaluated using 13 fold changes (log_2_ scale) from each of 13 high-quality RNA-seq datasets. Relative uncertainty of characterization was used as characterization uncertainty (*u*_*char*_), which can be expressed as equation (1) as follows:

1 $${u}_{ch{ar}}=\frac{\sqrt{\frac{\mathop{\sum }\nolimits_{i=1}^{n}{({x}_{i}-\bar{x})}^{2}}{(n-1)\times n}}}{\bar{x}}$$

where *n* is number of measurements in the sample; *x*_*i*_ is measurement value of *i*th time; and $\bar{x}$ is average value of *x* across *n* times.

Second, sample inhomogeneity ${u}_{{bb}}$ was evaluated using RNA-seq datasets. ${u}_{{bb}}$ can be expressed as equation as equations (2) and (3):

When $${s}_{1}^{2} > {s}_{2}^{2}$$,

2 $${u}_{{bb}}=\sqrt{\frac{{s}_{1}^{2}-{s}_{2}^{2}}{n}}$$

When $${s}_{1}^{2} < {s}_{2}^{2}$$,

3 $${u}_{{bb}}=\sqrt{\frac{{s}_{2}^{2}}{n}}\times \sqrt[4]{\frac{2}{{v}_{{s}_{2}^{2}}}}$$

where ${s}_{1}^{2}$ is between-unit variation; ${s}_{2}^{2}$ is within-unit variation; ${v}_{{s}_{2}^{2}}$ is degree of freedom of ${s}_{2}^{2}$; and *n* is number of between-unit measurements.

Third, long-term instability (*u*_*s*_) was evaluated based on RNA quality RIN across 20 months, which can be expressed as equation (4)

4 $${u}_{s}=t\times \sqrt{\frac{\mathop{\sum }\nolimits_{i=1}^{n}{(\,{y}_{i}-{b}_{0}-{b}_{1}{x}_{i})}^{2}}{(n-2)\times {\sum }_{i=1}^{n}{\left({x}_{i}-\bar{x}\right)}^{2}}}$$

where b_0_ and b_1_ are the intercept and slope of linear regression line between *x*_*i*_ (month) and *y* (RIN); *t* is time (month); and *n* is number of observations. Short-term instability might be negligible, because reference materials are recommended to be transported using dry ice.

Fourth, a combined uncertainty (*u*_*c*_) should consider all uncertainty described above, which can be expressed as equation (5):

5 $${u}_{c}=\sqrt{{{u}_{{char}}}^{2}+{u}_{{bb}}^{2}+{u}_{s}^{2}}$$

Finally, an extended uncertainty (*U*) can be expressed as equation (6):

6 $$U=k\times {u}_{c}$$

where *k* is a constant value. Here, *k* = 2 was applied for 95% confidence level.

### Performance metrics

Performance metrics, including SNR, RC with reference datasets, RMSE of differences with reference datasets and MCC of DEGs, were developed to evaluate the quality of RNA-seq data at expression level before a total score was calculated.

#### SNR

SNR is a measurement used in science and engineering. SNR is defined as the ratio of the power of a signal to the power of noise and is often expressed in decibels (https://en.wikipedia.org/wiki/Signal-to-noise_ratio). In this study, the average distances representing the intrinsic ‘differences’ among distinct biological sample groups are regarded as the signal, whereas the average distances among technical replicates of the same sample group are regarded as noise.

To identify an effective way to calculate the SNR values, we evaluated the performances of SNR values calculated by five different algorithms depending on whether the sample–sample ‘distance’ (signal or noise) is calculated based on the original feature space or the dimensionality-reduced space and how the distance was calculated. For the original feature space, the distance was calculated in three different ways: Euclidean distance or (1 − Pearson correlation coefficient) with all features considered simultaneously as a vector to represent a sample (abbreviated as OriAll_EucDist or OriAll_1-Cor, respectively) and the median of the Euclidean distances across all features when each single feature is separately used to represent a sample (OriSingle_MedianEucDist). For the dimensionality-reduced space, the Euclidean distance was calculated using the coordinates of a sample in the PC space from either *t*-distributed stochastic neighbor embedding (tSNE) or PCA, abbreviated as ReducedDim_tSNE and ReducedDim_PCA, respectively. The numbers of PCs used in calculating SNR were then determined. We decided to use the first two components in PCA to calculate SNR values in correspondence with visualization in PCA plots.

Therefore, SNR is defined as equation (7):

7 $$\begin{array}{l}{SNR}=10\times {\log }_{10}\left(\frac{m\times \left({n}\atop{2}\right)}{\left({m}\atop{2}\right)\times n\times n}\right.\\ \left.\qquad \quad\times \frac{\mathop{\sum }\nolimits_{x=1}^{m-1}\mathop{\sum }\nolimits_{y=x+1}^{m}\mathop{\sum }\nolimits_{i=1}^{n}\mathop{\sum }\nolimits_{j=1}^{n}\mathop{\sum }\nolimits_{p=1}^{2}{W}_{p}{\big(P{C}_{p,i,x}-P{C}_{p,\,j,y}\big)}^{2}}{\mathop{\sum }\nolimits_{x=1}^{m}\mathop{\sum }\nolimits_{i=1}^{n}\mathop{\sum }\nolimits_{j=i+1}^{n}\mathop{\sum }\nolimits_{p=1}^{2}{W}_{p}{\big(P{C}_{p,i,x}-P{C}_{p,\,j,x}\big)}^{2}}\right)\end{array}$$

where *m* is the number of sample groups, and *n* is the number of replicates in each sample group. *W*_*p*_ represents the *p*th PC of variances. $P{C}_{p,i,x},P{C}_{p,\,j,x}$ and $P{C}_{p,\,j,y}$ represent the *p*th component values of replicate *i* and replicate *j* in sample group *x* or sample group *y*, respectively.

A standard sample set consisted of 12 tubes with each representing one of the three replicates of the four RNA reference materials. Therefore, a typical SNR in the study was the ratio of the average distances between different biological groups (9 × 12/2 = 54) to the average distances between technical replicates of the same groups (2 × 3 × 4/2 = 12). The distribution of intra-batch SNR values from 21 RNA-seq datasets was used to identify a threshold of 12 (mean − s.d.), indicative of high discriminating power.

#### RC

RC with reference datasets was calculated based on the Pearson correlation coefficient between the ratio-based expression levels of a dataset for a given pair of groups and the corresponding reference fold change values. It is referred to as the ‘relative correlation with reference datasets’ metric, representing the numerical consistency of the ratio-based expression profiles. To improve reliability, the mean of the three replicates of each sample group was calculated before performing ratio-based expression analysis. Fold changes were transformed using log_2_ scaling.

#### RMSE

RMSE was calculated using fold changes between a test dataset for a given pair of samples and the corresponding ratio-based reference datasets, representing the average distances of ratio-based expression profiles. Fold changes were transformed using log_2_ scaling. It was implemented using the rmse function from the Metrics package^77^.

#### MCC

MCC is a widely used statistic in the field of bioinformatics and machine learning, which combines test sensitivity and specificity^20,78^. In this study, we used MCC to measure the consistency of DEGs detected from a dataset for a given pair of samples with those from the reference DEGs or ‘MCC of DEGs’. Reference DEGs and non-DEGs as true-positive (TP) and true-negative (TN) sets were integrated by consensus voting. When DEGs and non-DEGs of a given dataset were identified, the number of TP, TN, false positive (FP) and false negative (FN) could be calculated. MCC is computed using equation (8):

8 $${MCC}=\frac{{\rm{TP}}\times {\rm{TN}}-{\rm{FP}}\times {\rm{FN}}}{\sqrt{({TP}+{FP})({TP}+{FN})({TN}+{FP})({TN}+{FN})}}$$

### Total quality score

The total quality score is calculated to measure the overall quality of a dataset generated from a laboratory for its effectiveness in quantifying the transcriptomic differences among the four Quartet RNA reference materials by summarizing reference dataset-independent quality measurement (SNR) and reference dataset-dependent quality measurement (RC). The total quality score is expressed as the geometrical mean of SNR and RC.

### Cross-validation of reference-based quality metrics

To examine if the lower RC, higher RMSE or lower MCC and MCC for the low-quality batches were caused by their exclusion from creating the reference datasets, we performed 30 times of cross-validation test. In brief, in one cross-validation, we randomly selected 13 batches from the 21 batches to create (‘train’) the reference datasets, which were then used to calculate quality measurements for all the 21 batches. Both high-quality and low-quality batches might be randomly included or excluded from ‘training the reference datasets’, either as training or validation sets.

### Co-expression analysis

Co-expression network was constructed using the R package WGCNA version 1.71 (ref. ^43^) using absolute expression and ratio-based expression profiles of 13 batches with high quality, respectively. Genes with the highest variations (*n* = 10,000) were used for conducting co-expression network. Modules were then identified with a dynamic tree-cutting algorithm with a minimum module size of 50. Modules were named in color. Thirteen (13) modules were identified based on absolute expressions, including turquoise (*n* = 4,633), blue (*n* = 933), brown (*n* = 753), yellow (*n* = 728), green (*n* = 527) and so on. Moreover, eight modules were identified based on ratio-based expressions, including turquoise (*n* = 2,368), blue (*n* = 1,777), brown (*n* = 1,508), yellow (*n* = 741), green (*n* = 477), red (*n* = 229), black (*n* = 177) and pink (*n* = 133). PCA and functional analysis of each module were conducted.

Functional enrichment analyses of each module were conducted based on GO terms and were conducted with the R/Bioconductor package clusterProfiler version 4.2.2, with a Benjamini–Hochberg correction and an adjusted *P* value cutoff of 0.05 (ref. ^64^).

### RT–qPCR

Primers of 83 genes were designed using online Primers-BLAST of the National Center for Biotechnology Information based on the RNA sequences, and the PCR method of reference gene (*C1ORF43*) was established previously. Primers were synthesized by Beijing Liuhe Huada Gene Technology Co. Ltd. Sequences of primers are listed in Supplementary Table 8.

RT–qPCR reactions were performed in two steps. First, reverse transcription was carried out using 2 μl of RNA mixed with 4 μl of 5× PrimeScript IV cDNA Synthesis Mix (Takara, code no. 6215A) containing PrimeScript IV RTase, RNase Inhibitor, Oligo dT Primer, dNTP Mixture buffer and 1 μl of random 6mers and nuclease-free water up to 20-μl final reaction volume. This reaction mixture was incubated at 30 °C for 10 min and then for 15 min at 42 °C and finally for 5 min at 95 °C for termination. Second, cDNA obtained in the previous step was used as template for qPCR. The qPCR reactions were carried out using UltraSYBR Mixture (Low ROX) (CWBIO, CW2601M) containing 2 μl of cDNA and 0.4 μl of each forward and reverse primers (final concentration of 200 nM) in a 20-μl final volume reaction. The qPCR was performed on a Roche 480 qPCR System using the following cycling conditions: 10 min at 95 °C, followed by 45 cycles of 15 s at 95 °C and 1 min at 60 °C. Three replicates per sample per gene were conducted for eliminating random variations.

The comparative cycle threshold (Ct) method (ΔΔCt method) was used to calculate the fold differences for the three sample pairs (D5/D6, F7/D6 and M8/D6) with housekeeping gene *C1ORF43* as endogenous control. For the qPCR data, a gene is called DEG when the Student’s *t*-test *P* value < 0.05 and fold change ≥2 or ≤0.5.

### ddPCR

DEGs identified in reference datasets and RT–qPCR were further validated using ddPCR. The same sequences of primers used for RT–qPCR were used for ddPCR (Supplementary Table 8). The ddPCR reaction was performed in a QX200 Droplet Digital PCR System (Bio-Rad) according to the manufacturer’s instructions. Each test was prepared in a total of 20-μl volume of the reaction mixture, comprising 10 μl of EvaGreen Supermix (Bio-Rad), 2 µl of forward and reverse primers, 2 μl of cDNA templates and 6 μl of RNase-free ddH_2_O. Samples and 70 μl of droplet generation oil were then placed into a Droplet Generator (Bio-Rad). Droplets (40 μl) were transferred to a 96-well PCR plate. The PCR reactions were performed using the following cycling conditions: pre-denature for one cycle at 95 °C for 5 min; denature for 40 cycles at 95 °C for 30 s; and anneal and extend for 40 cycles at 60 °C for 1 min. After the cycles, a signal stabilization step of 4 °C for 5 min and 90 °C for 5 min was conducted. The signals were read by a Droplet Reader (Bio-Rad). Each reaction was performed in duplicate.

### Flow cytometry

Immortalized B-lymphoblastoid cells were centrifuged at 500*g* for 10 min at room temperature. Flick or aspirate to remove supernatant, and wash cells with 2 ml of PBS at 500*g* for 5 min at room temperature. For the sample stain, 1 × 10^6^ cells were resuspended in 100 μl of PBS with 2% FBS (FACS buffer) and stained with antibody cocktail for 15 min at room temperature in the dark. After surface staining, cells were washed twice with 2 ml of PBS at 500*g* for 5 min at room temperature. After the final wash, cells were resuspended in 250 μl of 1% paraformaldehyde (PFA).

The following antibodies were used for cell surface staining: PE mouse anti-human IgA (Miltenyi Biotec, 130-114-002, clone IS11-8E10), PE-Cy7 mouse anti-human IgD (BD Biosciences, 561314, clone IA6-2), Alexa Fluor 700 mouse anti-human IgG (BD Biosciences, 561296, clone G18-145) and Brilliant Violet 605 (BV605) mouse anti-human IgM (BD Biosciences, 562977, clone G20-127). PE mouse anti-human IgA was verified by the vendor, Miltenyi Biotec, including specificity, sensitivity and fixation. PE-Cy7 mouse anti-human IgD, AF700 mouse anti-human IgG and BV605 mouse anti-human IgM were validated by our previous study by flow cytometry^79^. Flow cytometric analyses were performed on CytoFLEX LX (Beckman Counter), and data were analyzed with FlowJo version 10.7.2 software (BD Biosciences).

The representative gating strategy for flow cytometry experiments assessing LCLs is shown in Supplementary Fig. 17. For the exclusion of non-single events, cross-check the forward scatter (FSC) signal for its area (A) versus height (H) and width (W) characteristics. Immortalized B-lymphoblastoid cells were gated on the FSC-A versus SSC-A dot plot. Furthermore, IgD^+^ cells, IgM^+^ cells, IgG^+^ cells and IgA^+^ cells in LCLs were identified based on their expression levels of surface membrane immunoglobulins.

### LC–MS/MS-based proteomics

MS-based data-dependent acquisition (DDA) proteomics dataset from Quartet protein reference materials was used for cross-omics validation. Detailed description of sample preparation and data generation was provided by Zheng et al.^52^. In brief, large quantities of Quartet peptide reference materials (lot: 20200616) were generated from the same generation of LCLs used in Quartet RNA reference materials. LC–MS/MS-based proteomics data (4 groups × 3 replicates) were then generated in a laboratory (code: NVG) using a Q Exactive HF-X mass spectrometer (Thermo Fisher Scientific).

Peptide and protein identification and quantification were conducted using Proteome Discoverer 2.2 (PD 2.2, Thermo Fisher Scientific) based on the human reference database UniProt (http://www.uniprot.org). Proteins with at least one unique peptide with 1% FDR at the peptide level were retained for further analysis. Protein quantification was normalized using the fraction of total (FOT). The fraction of total was multiplied by 10^5^ for ease of presentation.

### Biological classifications from published datasets

We used publicly available datasets to examine the extent of biological differences with four ‘intrinsic’ biological classification groups from published datasets^34–36^. Expression profiles in FPKM of four subtypes of TNBCs with different therapeutic actions were downloaded from previous publication^34^, including basal-like and immune-suppressed (*n* = 124), luminal androgen receptor (*n* = 75), immunomodulatory subtype (*n* = 77) and mesenchymal-like subtype (*n* = 60). Expression profiles in FPKM of the four molecular subtypes of breast cancer were downloaded from the Genomic Data Commons (GDC) Data Portal^35^, including luminal A (*n* = 420), luminal B (*n* = 174), basal-like (*n* = 140) and Her2-enriched (*n* = 65). Expression profiles in FPKM from four cancer types with distinct tissue types were also downloaded from the GDC Data Portal^35^, including brain cancer (*n* = 74), breast cancer (*n* = 77), kidney cancer (*n* = 67) and lung cancer (*n* = 66). Expression profiles in count from four normal tissue types were obtained from GTEx version 8, including brain (*n* = 100), breast (*n* = 100), kidney (*n* = 89) and lung (*n* = 100)^36^. Count data were normalized to CPM using the limma version 3.50.0 (ref. ^73^) package. Three samples from each clinical subtype or biological group were randomly selected for differential expression analysis to eliminate the effect of number of samples used for analysis. To eliminate selection biases, this process was repeated 20 times. A gene was considered as a DEG when *t*-test *P* < 0.05 and fold change ≥2 or ≤0.5.

### Statistical analysis

All statistical analyses were performed using R statistical software version 4.1.2 (https://www.r-project.org). PCA was conducted with the univariance scaling, using the prcomp function. Hierarchical clustering analysis (HCA) was performed using the R package pheatmap version 1.0.12 (https://rdrr.io/cran/pheatmap/). Data visualization was implemented using the R package ggplot2 version 3.3.5 (https://ggplot2.tidyverse.org/), GGally version 2.1.2 (http://ggobi.github.io/ggally/) and ggsci version 2.9 (https://github.com/nanxstats/ggsci).

### Reporting summary

Further information on research design is available in the Nature Portfolio Reporting Summary linked to this article.

## Online content

Any methods, additional references, Nature Portfolio reporting summaries, source data, extended data, supplementary information, acknowledgements, peer review information; details of author contributions and competing interests; and statements of data and code availability are available at 10.1038/s41587-023-01867-9.

## Supplementary information

Supplementary Information Supplementary Figs. 1–18.

Reporting Summary

Supplementary Tables 1–10.

## Data Availability

The raw sequence data and gene expression data reported in this paper have been deposited in the Genome Sequence Archive (GSA) (accession number: HRA001859)^80^ and the Open Archive for Miscellaneous Data (OMIX) (accession number: OMIX002254)^81^ of the China National Center for Bioinformation. Moreover, we developed the Quartet Data Portal (http://chinese-quartet.org) for the community to access and share the Quartet multi-omics resources.

## Code availability

The source codes for the data analyses are available at Zenodo^82,83^.
