# Author Correction: Quartet RNA reference materials improve the quality of transcriptomic data through ratio-based profiling

**DOI:** 10.1038/s41587-023-02008-y

**Published:** 2023-10-02

**Authors:** Ying Yu, Wanwan Hou, Yaqing Liu, Haiyan Wang, Lianhua Dong, Yuanbang Mai, Qingwang Chen, Zhihui Li, Shanyue Sun, Jingcheng Yang, Zehui Cao, Peipei Zhang, Yi Zi, Ruimei Liu, Jian Gao, Naixin Zhang, Jingjing Li, Luyao Ren, He Jiang, Jun Shang, Sibo Zhu, Xiaolin Wang, Tao Qing, Ding Bao, Bingying Li, Bin Li, Chen Suo, Yan Pi, Xia Wang, Fangping Dai, Andreas Scherer, Pirkko Mattila, Jinxiong Han, Lijun Zhang, Hui Jiang, Danielle Thierry-Mieg, Jean Thierry-Mieg, Wenming Xiao, Huixiao Hong, Weida Tong, Jing Wang, Jinming Li, Xiang Fang, Li Jin, Joshua Xu, Feng Qian, Rui Zhang, Leming Shi, Yuanting Zheng

**Affiliations:** 1 State Key Laboratory of Genetic Engineering, School of Life Sciences and Human Phenome Institute, Shanghai Cancer Center, Fudan University, Shanghai, China; 2 National Institute of Metrology, Beijing, China; 3 Greater Bay Area Institute of Precision Medicine, Guangzhou, China; 4 Nextomics Biosciences Institute, Wuhan, China; 5 Genome Decoding Institute, Nantong, China; 6 Institute for Molecular Medicine Finland (FIMM), University of Helsinki, Helsinki, Finland; 7 EATRIS ERIC-European Infrastructure for Translational Medicine, Amsterdam, The Netherlands; 8 Nanjing Vazyme Biotech Co. Ltd., Nanjing, China; 9 MGI, BGI-Shenzhen, Shenzhen, China; 10 National Center for Biotechnology Information, National Library of Medicine, National Institutes of Health, Bethesda, MD USA; 11 Office of Oncologic Diseases, Office of New Drugs, Center for Drug Evaluation and Research, US Food and Drug Administration, Silver Spring, MD USA; 12 Division of Bioinformatics and Biostatistics, National Center for Toxicological Research, US Food and Drug Administration, Jefferson, AR USA; 13 National Center for Clinical Laboratories, Institute of Geriatric Medicine, Chinese Academy of Medical Sciences, Beijing Hospital, Beijing, China; 14 National Center of Gerontology, Beijing, China; 15 Shanghai Public Health Clinical Center, Fudan University, Shanghai, China; 16 International Human Phenome Institutes, Shanghai, China

**Keywords:** Quality control, Standardization, Transcriptomics

Correction to: *Nature Biotechnology* 10.1038/s41587-023-01867-9, published online 7 September 2023.

This paper was originally published under a standard Springer Nature license (© The Author(s), under exclusive licence to Springer Nature America, Inc.). It is now available as an open-access paper under a Creative Commons Attribution 4.0 International license, © The Author(s). The error has been corrected in the HTML and PDF versions of the article.

