# Supplementary material for: Quartet RNA reference materials improve the quality of transcriptomic data through ratio-based profiling
Source: Nat Biotechnol. 2023 Sep 7;42(7):1118–32. doi: 10.1038/s41587-023-01867-9 (PMC11251996; doi:10.1038/s41587-023-01867-9)
Supplement: Supplementary file 1 — Supplementary Figs. 1–18. [file 41587_2023_1867_MOESM1_ESM.pdf]

nature biotechnology

Article https://doi.org/10.1038/s41587-023-01867-9

Quartet RNA reference materials improve the quality of transcriptomic data through ratio-based profiling

In the format provided by the authors and unedited

Table of contents

Supplementary Fig. 1 | Quality of the Quartet RNA reference materials 2

Supplementary Fig. 2 | Stability assessment of the Quartet RNA reference materials 3

Supplementary Fig. 3 | Validation of analysis pipeline based on MAQC reference materials 5

Supplementary Fig. 4 | PCA plot for combining PolyA and RiboZero protocols. 6

Supplementary Fig. 5 | SNR is established for quality assessment across multiple samples 7

Supplementary Fig. 6 | PCA plots with SNR values across 21 RNA-seq batches 8

Supplementary Fig. 7 | Performance evaluation based on different gene quantification tools (RSEM or StringTie) in terms of intra-batch measurement. 9

Supplementary Fig. 8 | PCA plots with SNR values of two exemplary batches based on Percent Spliced In (PSI) values of alternative splicing events 10

Supplementary Fig. 9 | Assessment of homogeneity and long-term stability of the Quartet RNA reference materials 11

Supplementary Fig. 10 | Correlation of RNA-protein pairs under different criteria for selecting the number of features for plotting 12

Supplementary Fig. 11 | Concordance between different quality control metrics based on reference datasets 13

Supplementary Fig. 12 | PCA plots on RNAseq data of the MAQC RNA reference materials. 14

Supplementary Fig. 13 | PCA plots on RNAseq data before and after batch correction based on RSEM and/or StringTie tools. 15

Supplementary Fig. 14 | PCA plots on RNAseq data before (a) and after (b) batch correction based on normalized counts. 16

Supplementary Fig. 15 | Hierarchical clustering of the immortalized cell lines of the quartet family members 17

Supplementary Fig. 16 | Expression characteristics and enriched GO terms of co-expression modules based on log_2FPKM values. 18

Supplementary Fig. 17 | The representative gating strategy for flow cytometry experiments assessing immortalized B-lymphoblastoid cell lines. 19

Supplementary Fig. 18 | Hierarchical clustering of the whole-blood samples of the quartet family members 20

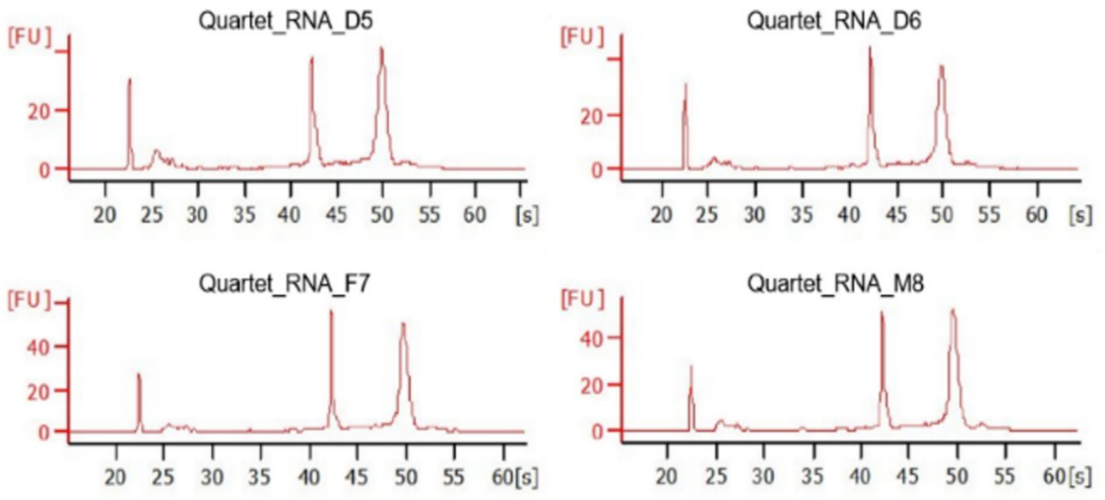

Supplementary Fig. 1 | Quality of the Quartet RNA reference materials

Electropherograms of the Quartet RNA reference materials reflecting RNA quality produced by Agilent 2100.

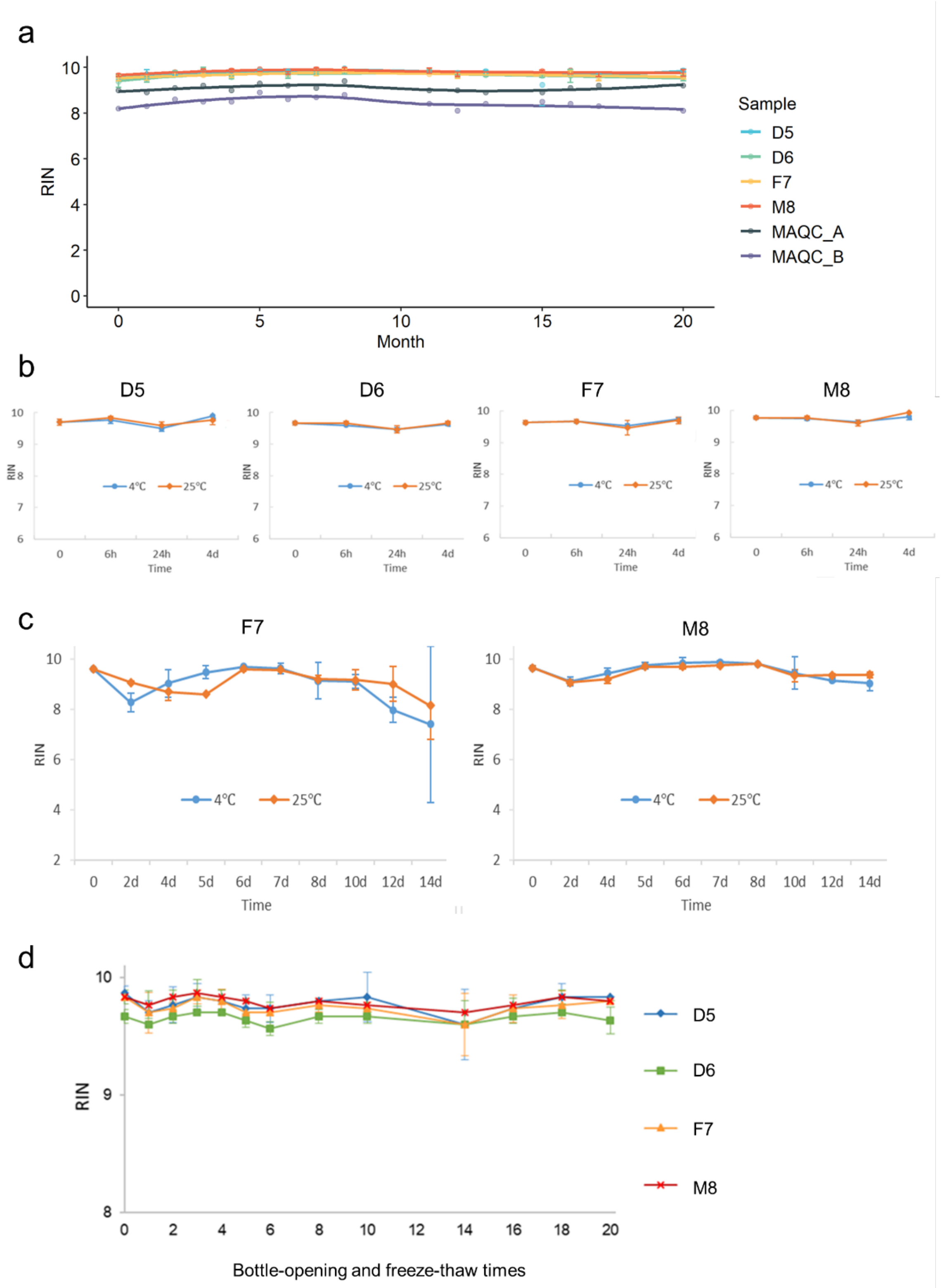

Supplementary Fig. 2 | Stability assessment of the Quartet RNA reference materials

(**a**) Distribution of RNA integrity number (RIN) values across 20 months during assessment. The Quartet RNA reference materials and two well-characterized and

commercialized RNA reference materials (MAQC A and B) were tested. (**b**) RIN values of the Quartet RNA reference materials across four days of storage at 4°C or 25°C. (**c**) RIN values of two reference materials (F7 and M8) across 14 days of storage at 4°C or 25°C. (**d**) RIN values of 20 times of bottle-opening and freeze-thaw cycle. Three replicates (n=3) of each RNA reference material were collected for each time point and for each condition. Data are presented as mean ± SD.

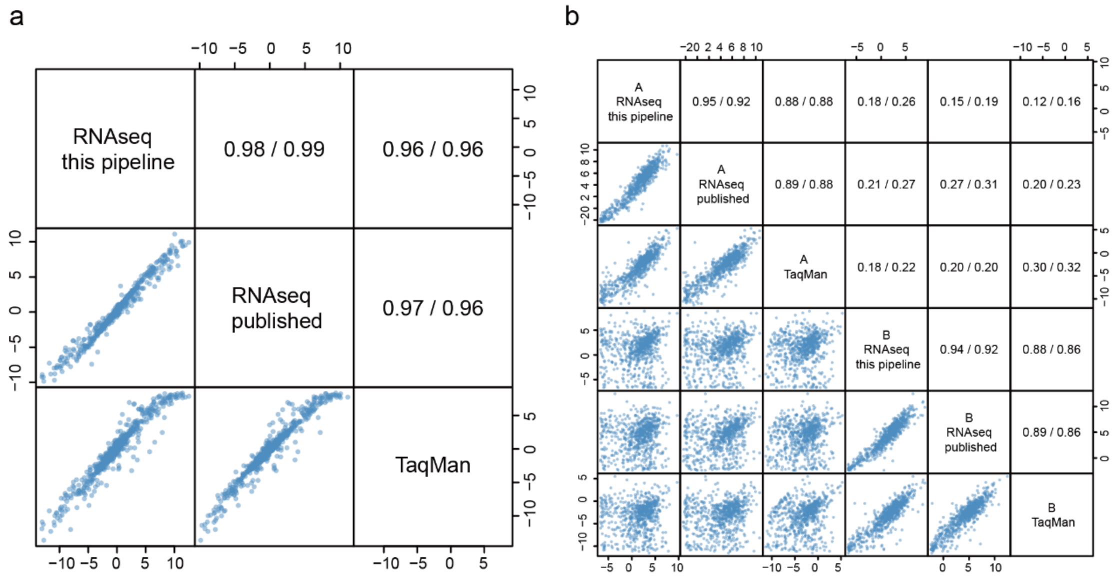

Supplementary Fig. 3 | Validation of analysis pipeline based on MAQC reference materials

The scatter plots compared the (a) log2-transrofmed fold differences or ratios (using MAQC A/B replicates) and (b) log2-transformed gene expression profiles of A and B from public data for validation of the analysis pipeline used in the study. Data included: (1) expression matrix generated based on analysis pipeline used in this study as the dataset for validation; (2) expression matrix obtained from publication[20] as a positive control dataset; and (3) TaqMan results downloaded from the public data[15] as "ground truth". Results from this pipeline and results from published dataset were based on the same RNA-seq data but with different analysis pipelines. Correlation coefficients based on ratios (A/B) were estimated for 725 selected genes that were commonly detected in RNA-seq and TaqMan assays. Good concordances were observed between RNA-seq and MAQC-I TaqMan assays. The results revealed that analysis pipelines in this study were reliable. Pearson/Spearman correlation coefficients were shown in the upper-right triangle.

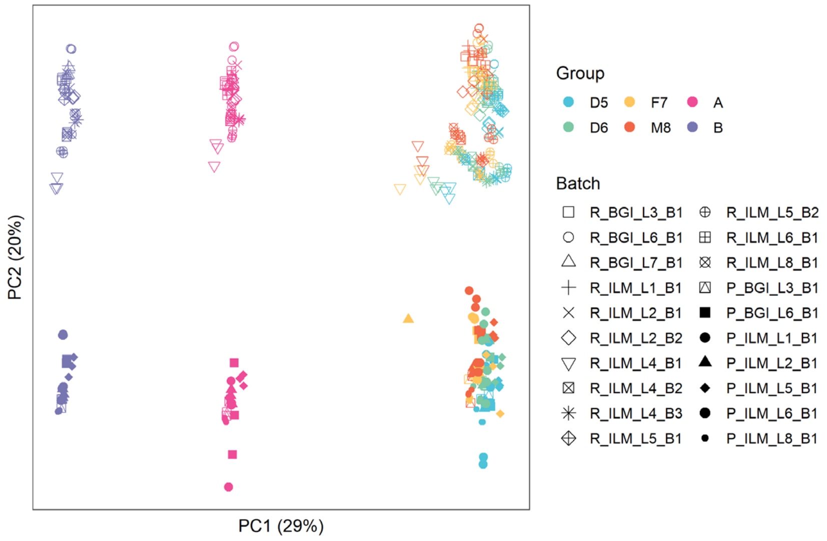

Supplementary Fig. 4 | PCA plot for combining PolyA and RiboZero protocols.

RNAseq data in log2-transformed FPKM from the Quartet and MAQC RNA reference materials (marked in colors) across 20 batches (marked in shapes) were used.

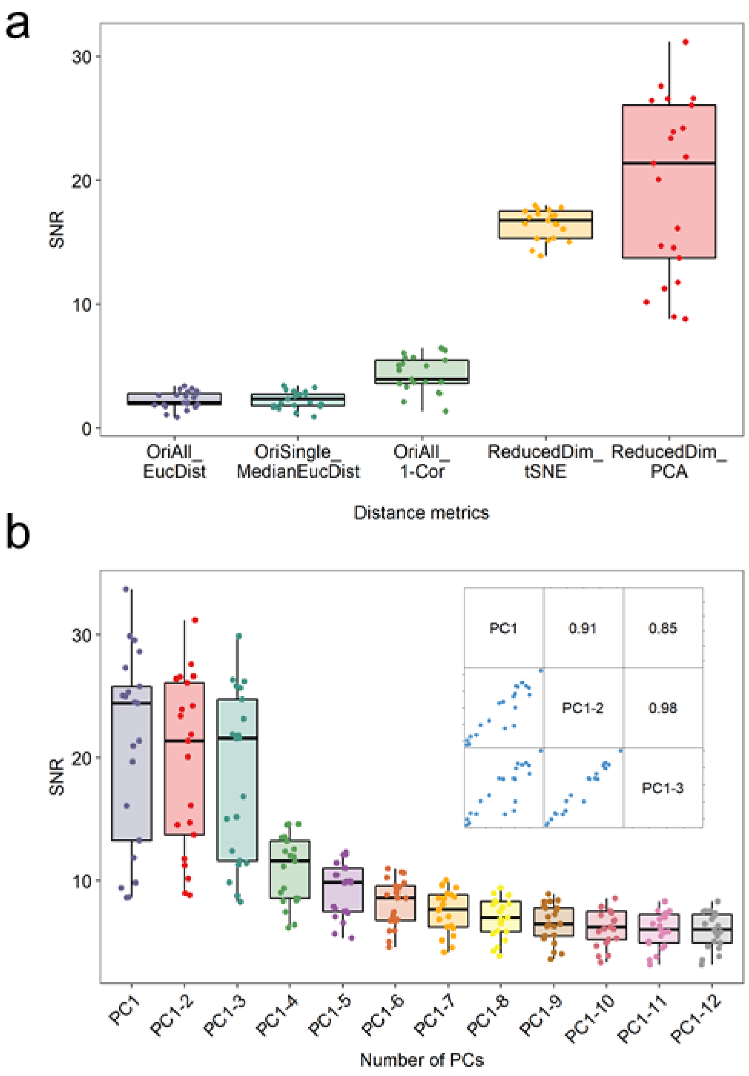

Supplementary Fig. 5 | SNR is established for quality assessment across multiple samples

(**a**) Boxplots of distribution of SNR values across 21 RNA-seq batches calculated by different distance metrics, including Euclidean distance (Dist), overall expression profiles (Expr), Pearson correlation coefficient (Cor), t-Distributed Stochastic Neighbor Embedding (tSNE), and principal components analysis (PCA). (**b**) Boxplots of distribution of SNR values across 21 RNA-seq batches calculated by using difference numbers of principal components.

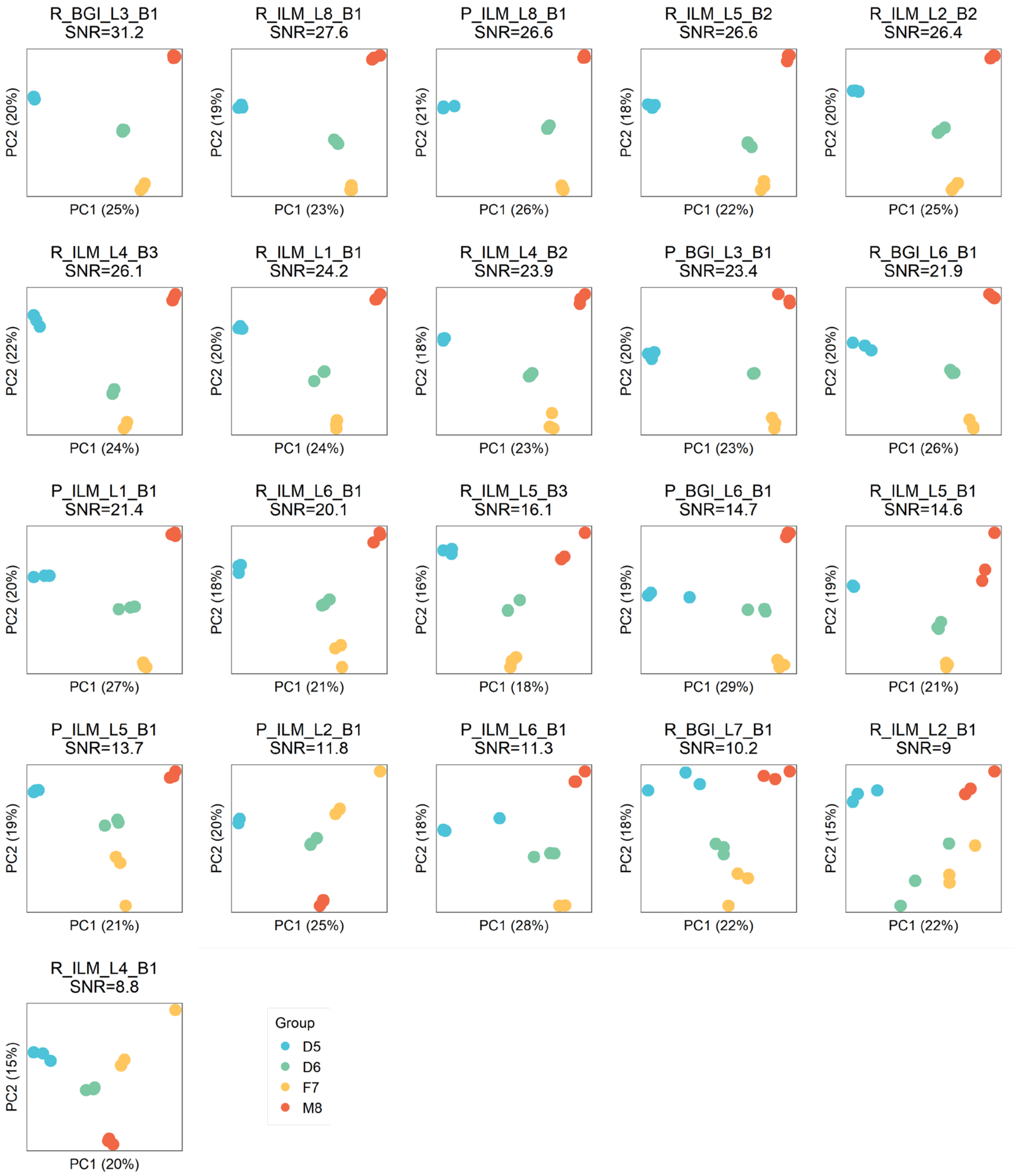

Supplementary Fig. 6 | PCA plots with SNR values across 21 RNA-seq batches

Scatter plots of principal component analysis (PCA) in each batch based on log_2FPKM normalized gene expression data. PCA plots were ordered by signal-to-noise ratio (SNR) values. Plots were color-coded by sample groups.

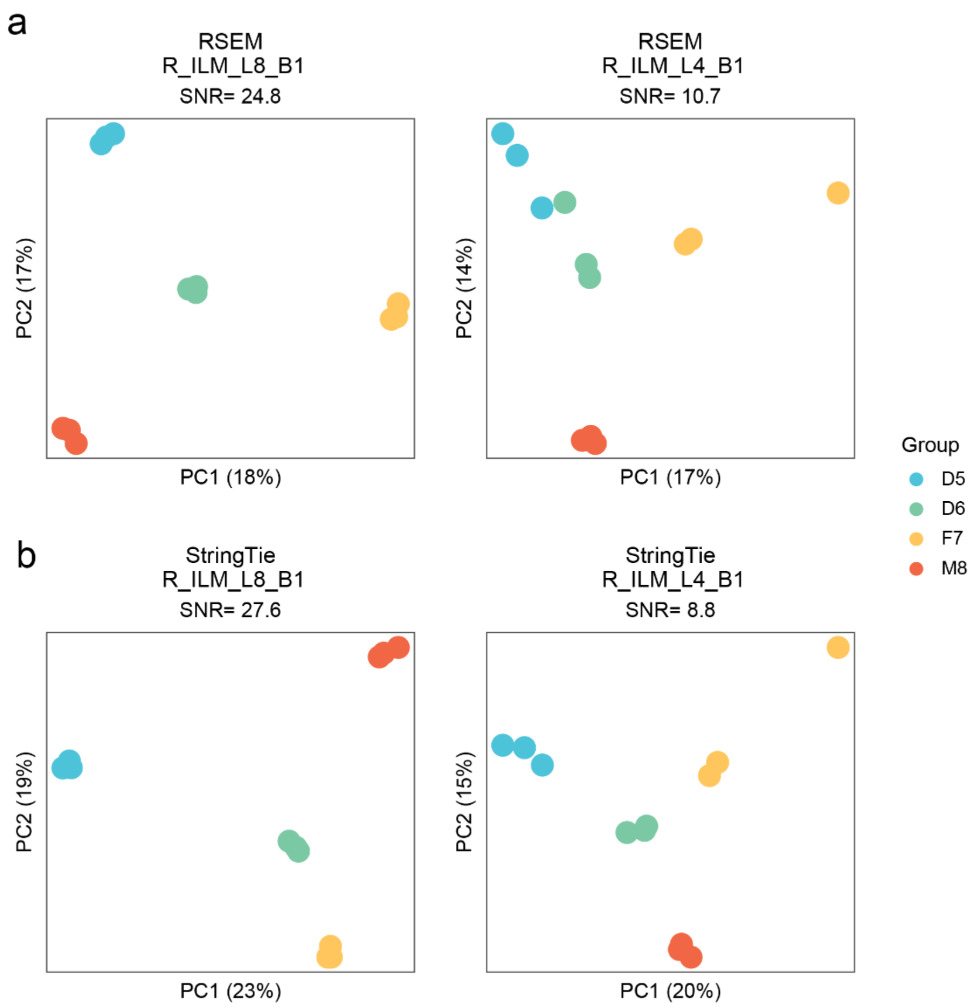

Supplementary Fig. 7 | Performance evaluation based on different gene quantification tools (RSEM or StringTie) in terms of intra-batch measurement.

PCA plots with SNR values of two exemplary batches based on RSEM and StringTie tools for quantification. RNAseq datasets from R_ILM_L8_B1 (as a high-quality batch) and R_ILM_L4_B1 (as a low-quality batch) were used for plotting.

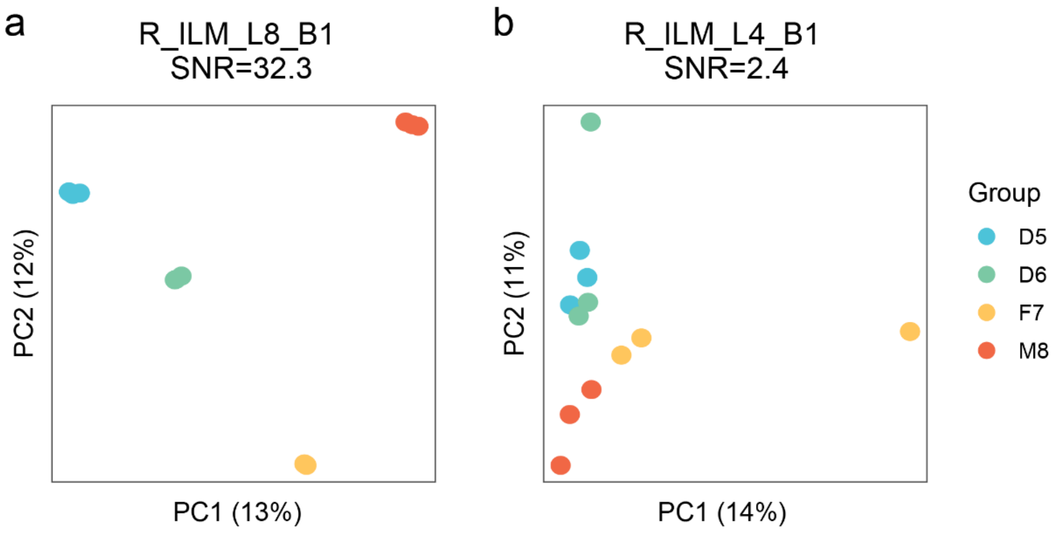

Supplementary Fig. 8 | PCA plots with SNR values of two exemplary batches based on Percent Spliced In (PSI) values of alternative splicing events

RNA-seq datasets from (**a**) R_ILM_L8_B1 (as a high-quality batch) and (**b**) R_ILM_L4_B1 (as a low-quality batch) were used for plotting.

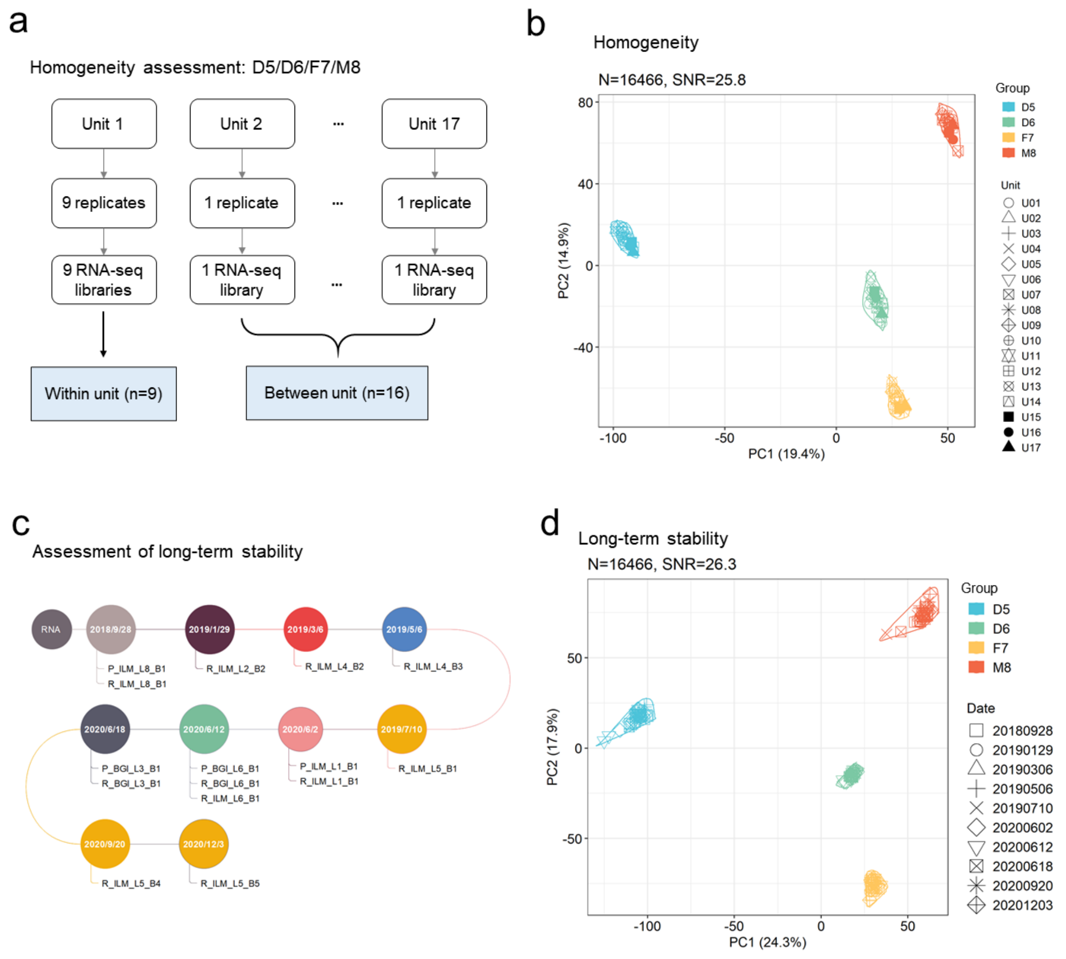

Supplementary Fig. 9 | Assessment of homogeneity and long-term stability of the Quartet RNA reference materials

(**a, c**) Schematic diagrams of assessing homogeneity (**a**) and long-term stability (**c**). (**b**) Scatter plot of principal component analysis (PCA) of expression profiles across 17 units for homogeneity assessment. Plot was color-coded by sample groups and shaped-coded by packaging (unit) ID. (**d**) Scatter plot of PCA of expression profiles from 15 batches of data generated from up to 26 months. Plot was color-coded by sample groups and shaped-coded by date of data generation. Ratio-based expressions were obtained by subtracting $\log_2$FPKM by the mean of $\log_2$FPKM of the three replicates of D6 in the same batch as used for plotting.

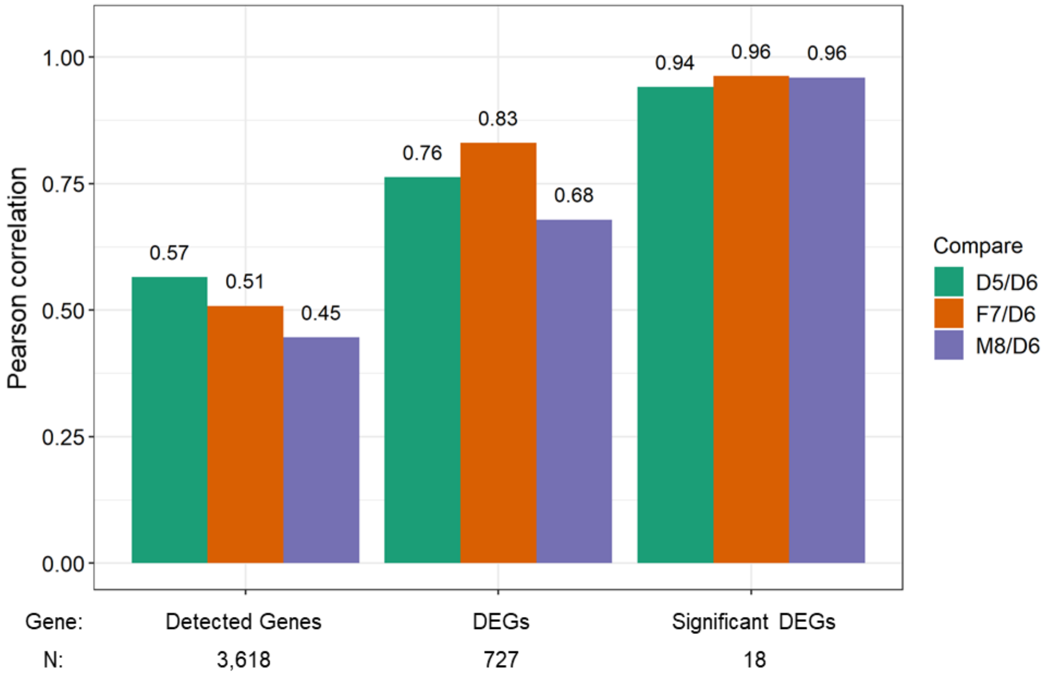

Supplementary Fig. 10 | Correlation of RNA-protein pairs under different criteria for selecting the number of features for plotting

A batch of LC-MS/MS proteomic dataset was used for cross-omics validation of RNA reference datasets. Pearson correlation coefficient was used to represent consistency between RNA and protein abundances. RNA-protein correlations varied considerably when different cut-offs were used to select genes for plotting. Three different cutoffs were used with increasing degree of stringency, including detectable genes, differentially expressed genes based on *p*-value <0.05, or significantly differentially expressed genes based on *p*-value <0.05 and fold change ≥ 2 or ≤ 0.5. Limma-based two-sided *p*-values were computed for RNA-seq data, while student's t-test two-sided *p*-values were computed for proteomics data. The exact fold changes and p-values of proteomics data could been viewed in **Supplementary Table 10**. The average numbers of genes across the three sample pairs were listed in the X-axis.

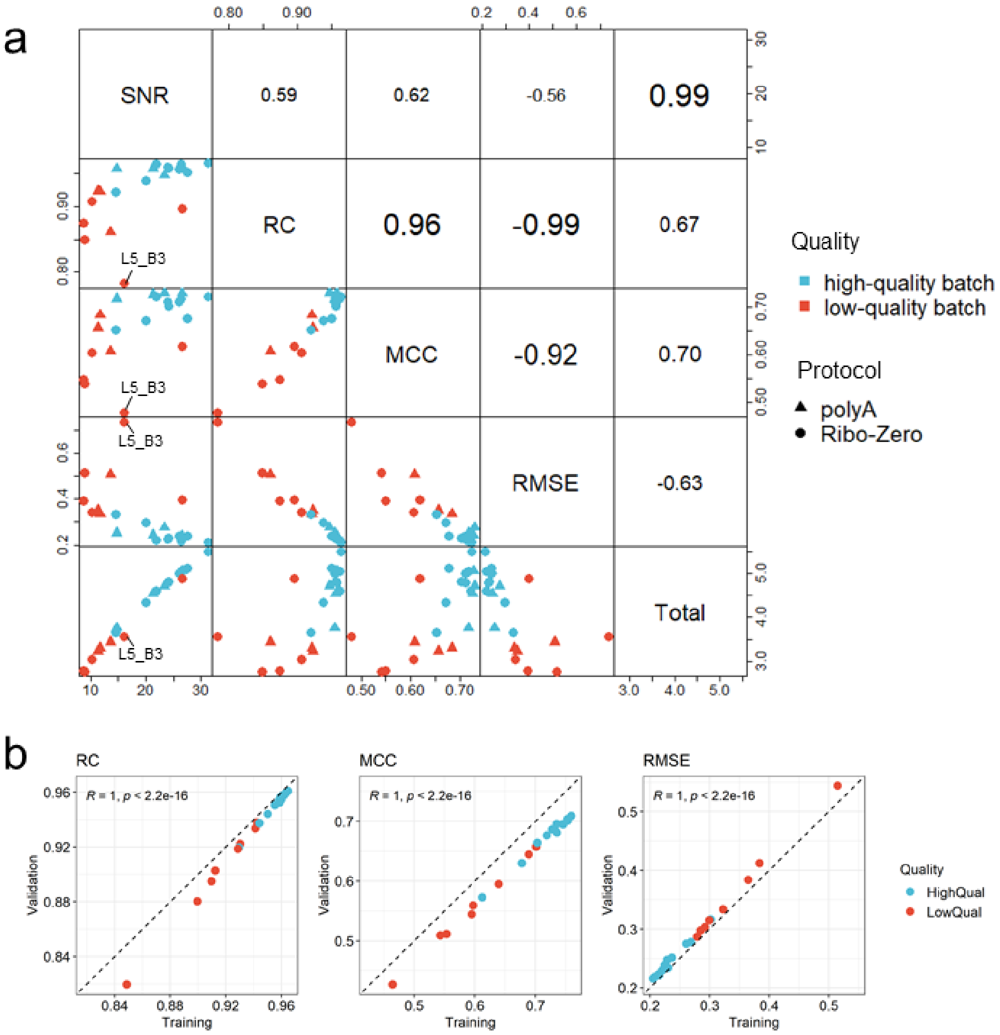

Supplementary Fig. 11 | Concordance between different quality control metrics based on reference datasets

Distribution of relative correlation with reference datasets (RC), MCC of DEGs, and root mean square error (RMSE) of the differences with reference datasets across 21 RNA-seq batches. (**b**) Average values of three quality metrics in trainng and validation sets in 30-time cross-validation test. The *p*-values in the plot are based on Pearson correlation test to assess whether a correlation is statistically significant (real).

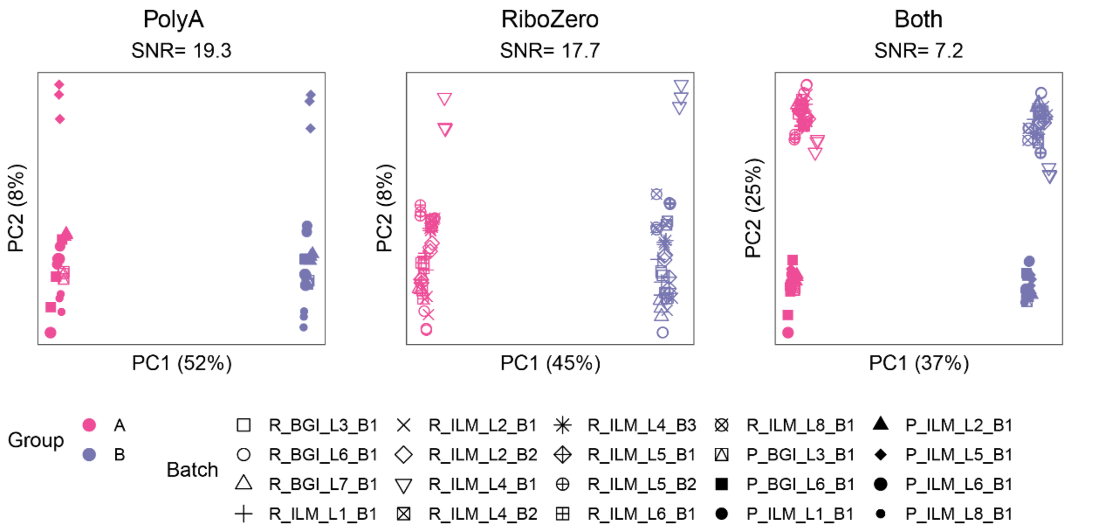

Supplementary Fig. 12 | PCA plots on RNAseq data of the MAQC RNA reference materials.

Expressions in log_2FPKM from a multi-batch RNAseq dataset of MAQC RNA reference materials were used, including 13 batches from RiboZero protocol and seven batches from PolyA protocol. Plots were color-coded by sample groups and shaped by batches.

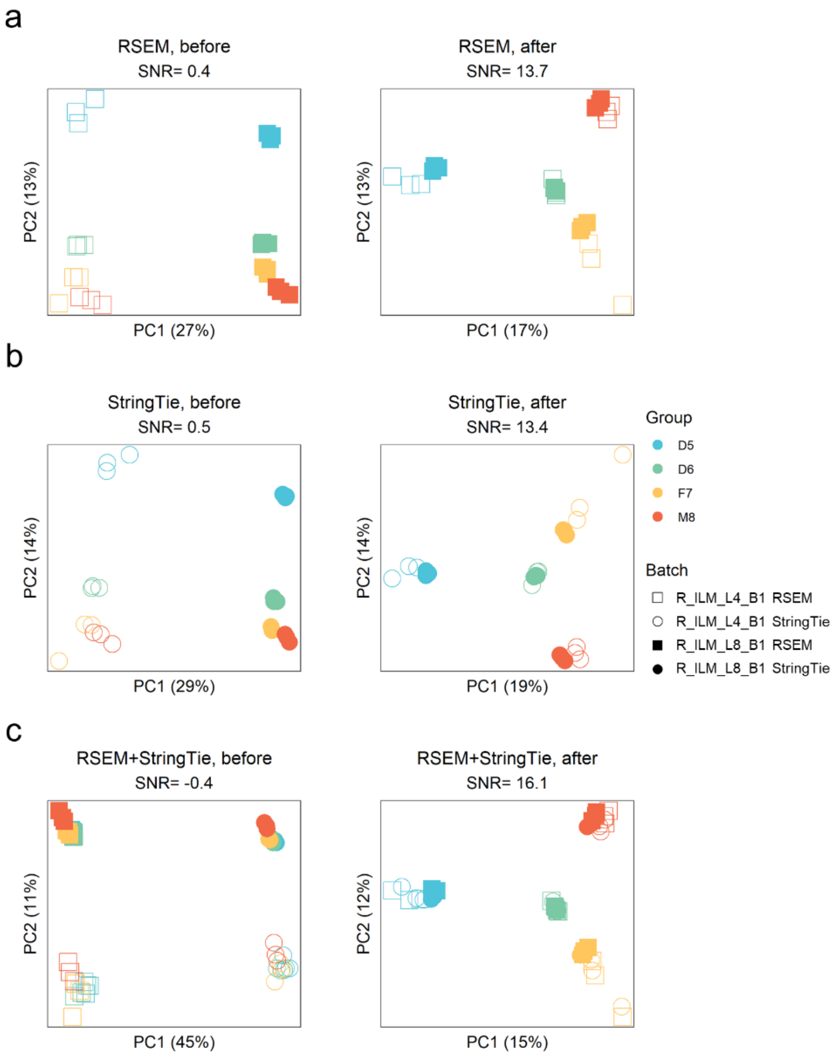

Supplementary Fig. 13 | PCA plots on RNAseq data before and after batch correction based on RSEM and/or StringTie tools.

Cross-batch integration of two exemplary batches were assessed, including (a) RSEM, (b) StringTie, and (c) combining of RSEM and StringTie. RNAseq datasets from R_ILM_L8_B1 (as a high-quality batch) and R_ILM_L4_B1 (as a low-quality batch) were used for plotting. Expressions in log2-transformed FPKM were used as before batch-correction datasets. Ratio-based expressions were obtained by subtracting log2-transformed FPKM by the mean of values of the three replicates of D6 in the same batch.

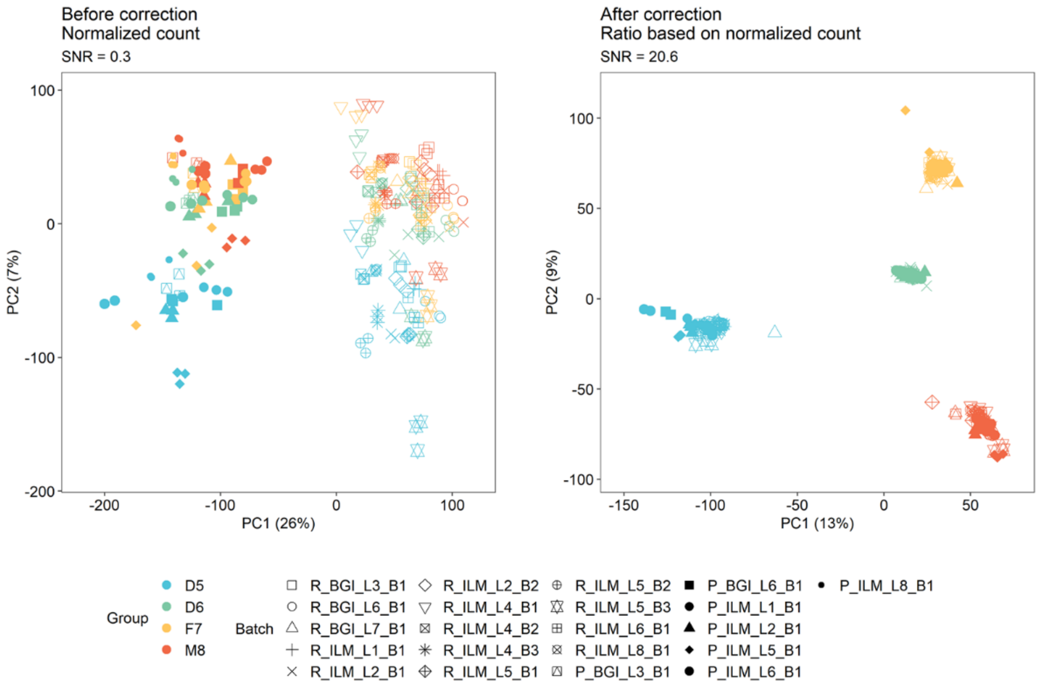

Supplementary Fig. 14 | PCA plots on RNAseq data before (a) and after (b) batch correction based on normalized counts.

Expressions in log2-transformed normalized counts were used as before batch-correction datasets. Ratio-based expressions were obtained by subtracting log2-transformed normalized counts by the mean of values of the three replicates of D6 in the same batch. Normalized counts were obtained using DESeq2 R/Bioconductor package. Plots were color-coded by sample groups and shaped by batches.

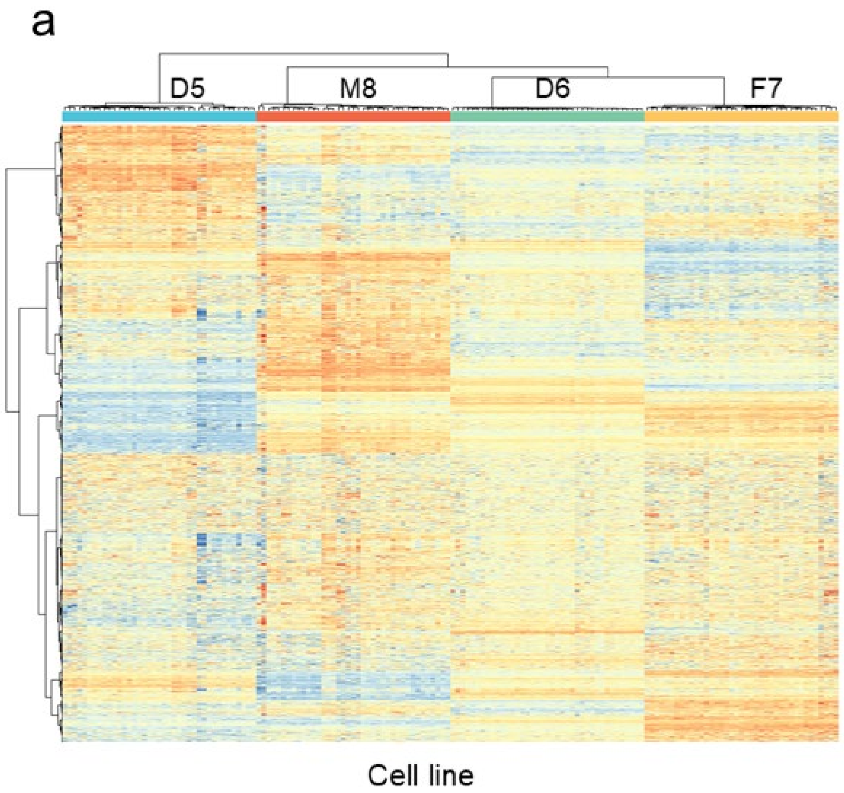

Supplementary Fig. 15 | Hierarchical clustering of the immortalized B-lymphoblastoid cell lines of the quartet family members

Hierarchical clustering based on 156 RNA-seq libraries from 13 batches with high quality derived from the four immortalized B-lymphoblastoid cell lines of the quartet family members. Ratio-based expressions of detected genes across all samples derived from immortalized cell lines were used (n=19,760).

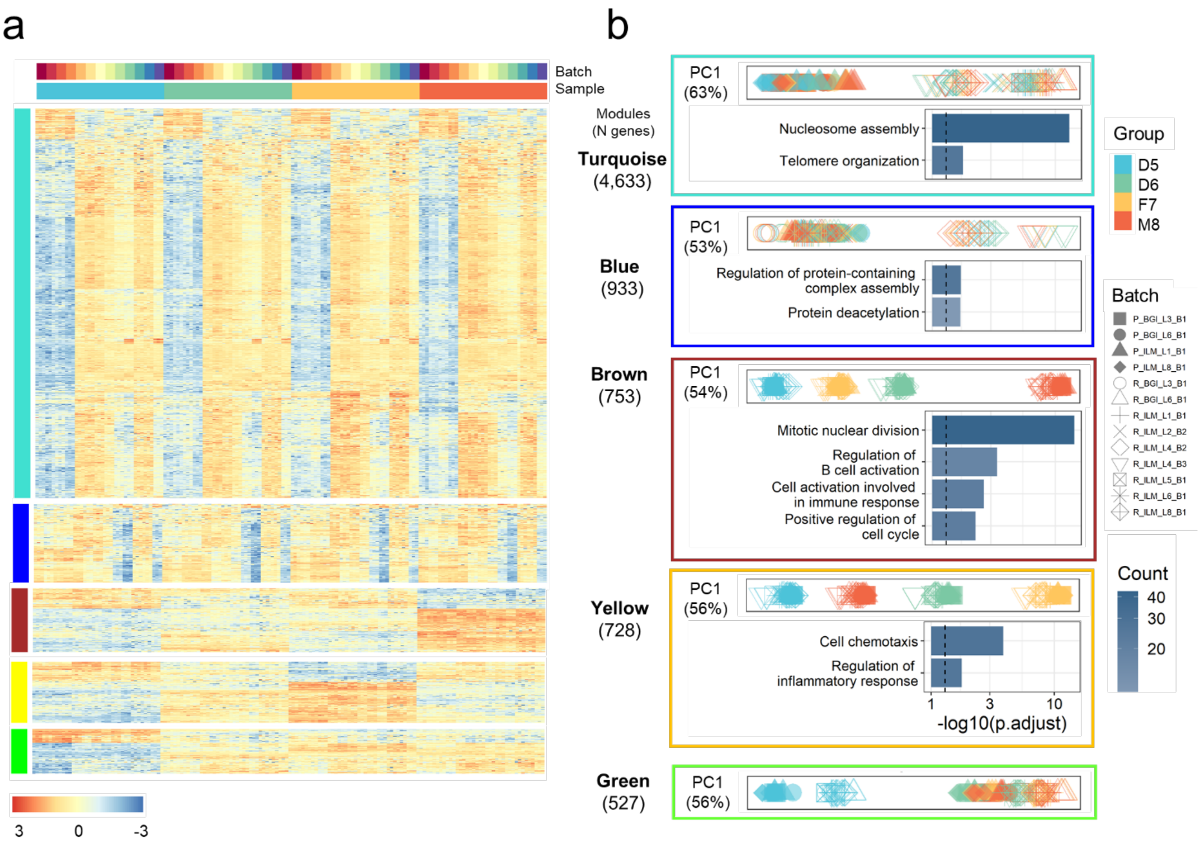

Supplementary Fig. 16 | Expression characteristics and enriched GO terms of co-expression modules based on log_2FPKM values.

(a) Expression profiles from five largest co-expression modules using data from 13 batches with high quality. (b) Distances of samples in PC1 space and list of GO terms enriched with genes in each corresponding module. Enriched GO terms were generated based on hypergeometric test using clusterProfiler. Benjamini & Hochberg (BH) adjusted p-value cutoff of 0.05 was set. PC plots were colored by sample groups and shaped by batches.

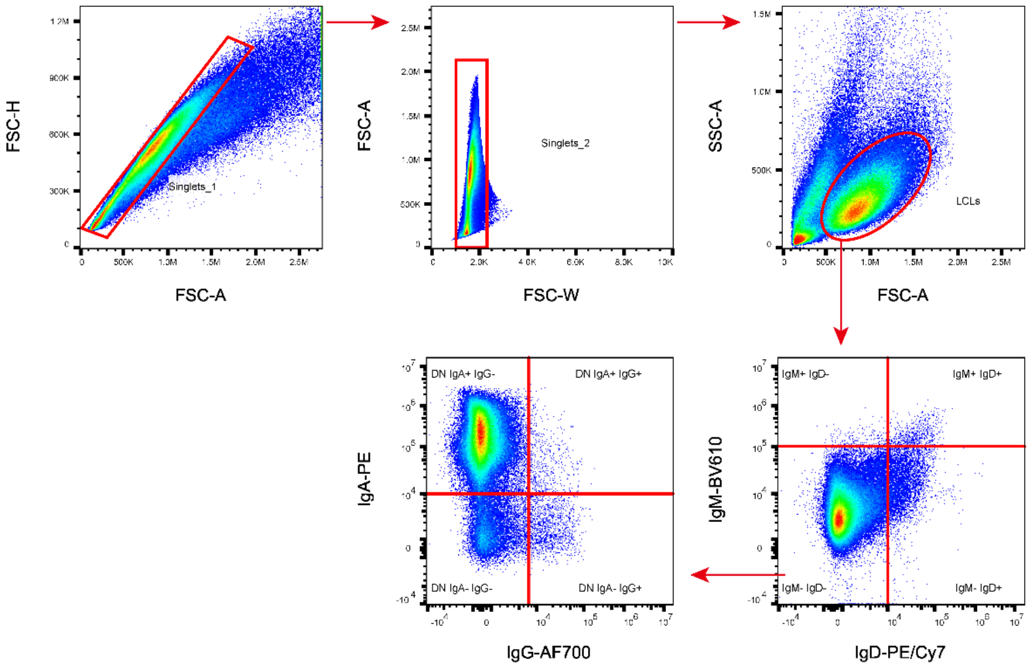

Supplementary Fig. 17 | The representative gating strategy for flow cytometry experiments assessing immortalized B-lymphoblastoid cell lines.

For the exclusion of non-single events, cross-check the forward scatter (FSC) signal for its area (A) versus height (H) and width (W) characteristics. Immortalized B-lymphoblastoid cells were gated on the FSC-A versus SSC-A dot plot. Furthermore, IgD+ cells, IgM+ cells, IgG+ cells, and IgA+ cells in immortalized B-lymphoblastoid cell lines were identified based on their expression levels of surface membrane immunoglobulins.

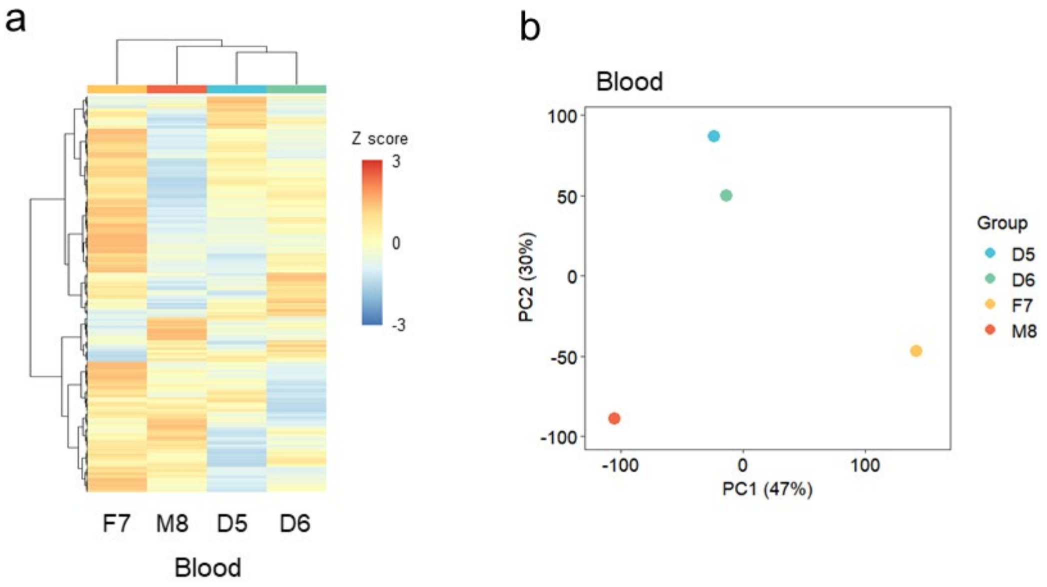

Supplementary Fig. 18 | Hierarchical clustering of the whole-blood samples of the quartet family members

(**a**) Hierarchical clustering (**a**) and PCA (**b**) based on transcriptomic profiles of four whole-blood samples from the quartet family members. The overall expression profiles of detected genes (n=22,623) from blood samples were used.
