# Supplementary material for: Quartet RNA reference materials improve the quality of transcriptomic data through ratio-based profiling
Source: Nat Biotechnol. 2023 Sep 7;42(7):1118–32. doi: 10.1038/s41587-023-01867-9 (PMC11251996; doi:10.1038/s41587-023-01867-9)
Supplement: Supplementary file 2 — Reporting Summary [file 41587_2023_1867_MOESM2_ESM.pdf]

Corresponding author(s): Joshua Xu, Feng Qian, Rui Zhang, Leming Shi, and Yuanting Zheng

Last updated by author(s): Jun 13, 2023

Reporting Summary

Nature Portfolio wishes to improve the reproducibility of the work that we publish. This form provides structure for consistency and transparency in reporting. For further information on Nature Portfolio policies, see our Editorial Policies and the Editorial Policy Checklist.

Statistics

For all statistical analyses, confirm that the following items are present in the figure legend, table legend, main text, or Methods section.

n/a	Confirmed	
☐	☒	The exact sample size (*n*) for each experimental group/condition, given as a discrete number and unit of measurement
☐	☒	A statement on whether measurements were taken from distinct samples or whether the same sample was measured repeatedly
☐	☒	The statistical test(s) used AND whether they are one- or two-sided<br>*Only common tests should be described solely by name; describe more complex techniques in the Methods section.*
☒	☐	A description of all covariates tested
☐	☒	A description of any assumptions or corrections, such as tests of normality and adjustment for multiple comparisons
☐	☒	A full description of the statistical parameters including central tendency (e.g. means) or other basic estimates (e.g. regression coefficient) AND variation (e.g. standard deviation) or associated estimates of uncertainty (e.g. confidence intervals)
☐	☒	For null hypothesis testing, the test statistic (e.g. *F*, *t*, *r*) with confidence intervals, effect sizes, degrees of freedom and *P* value noted<br>*Give P values as exact values whenever suitable.*
☒	☐	For Bayesian analysis, information on the choice of priors and Markov chain Monte Carlo settings
☒	☐	For hierarchical and complex designs, identification of the appropriate level for tests and full reporting of outcomes
☐	☒	Estimates of effect sizes (e.g. Cohen's *d*, Pearson's *r*), indicating how they were calculated

Our web collection on statistics for biologists contains articles on many of the points above.

Software and code

Policy information about availability of computer code

Data collection	Software: HISAT v2.1, SAMtools v1.3.1, StringTie v1.3.4, Ballgown v2.14.1, FastQC v0.11.5, FastQ Screen v0.12.0, Qualimap v2.0.0, MultiQC v1.8. The source codes for the data collection are available at Github (https://doi.org/10.5281/zenodo.8014752)
Data analysis	Software: R v4.1.2. R code for data analysis and figure generation is available at Github (https://doi.org/10.5281/zenodo.8014734)

For manuscripts utilizing custom algorithms or software that are central to the research but not yet described in published literature, software must be made available to editors and reviewers. We strongly encourage code deposition in a community repository (e.g. GitHub). See the Nature Portfolio guidelines for submitting code & software for further information.

Data

Policy information about availability of data

All manuscripts must include a data availability statement. This statement should provide the following information, where applicable:

- Accession codes, unique identifiers, or web links for publicly available datasets
- A description of any restrictions on data availability
- For clinical datasets or third party data, please ensure that the statement adheres to our policy

The raw sequence data and gene expression data reported in this paper have been deposited in the Genome Sequence Archive (GSA) (accession number: HRA001859, https://ngdc.cncb.ac.cn/gsa-human/browse/HRA001859) and Open Archive for Miscellaneous Data (OMIX) (accession number: OMIX002254, https://

ngdc.cncb.ac.cn/omix/release/OMIX002254). Moreover, we have developed the Quartet Data Portal (http://chinese-quartet.org) for the community to access and share the Quartet multiomics resources.

Human research participants

Policy information about studies involving human research participants and Sex and Gender in Research.

Reporting on sex and gender	We used blood samples from four participants from a Chinese Quartet family from the Fudan Taizhou Cohort, including father (F7), mother (M8) and monozygotic twin daughters (D5 and D6). Sex and gender of the participants are F7 (male), M8 (female), D5 (female) and D6 (female). Sex and gender information was determined based on self-reporting and DNA sequencing.
Population characteristics	All four participants are adults from Han Chinese. No additional covariates-relevant population characteristics were collected for each donor due to IRB approval restrictions.
Recruitment	The participants were recruited by advertisements in the Taizhou Longitudinal Study. We randomly selected a family with monozygotic twin daughters. No self-selection bias was expected to be introduced.
Ethics oversight	This study was approved by the Institutional Review Board (IRB) of the School of Life Sciences, Fudan University (BE2050). It was conducted under the principles of the Declaration of Helsinki.

Note that full information on the approval of the study protocol must also be provided in the manuscript.

Field-specific reporting

Please select the one below that is the best fit for your research. If you are not sure, read the appropriate sections before making your selection.

☒ Life sciences ☐ Behavioural & social sciences ☐ Ecological, evolutionary & environmental sciences

For a reference copy of the document with all sections, see nature.com/documents/nr-reporting-summary-flat.pdf

Life sciences study design

All studies must disclose on these points even when the disclosure is negative.

Sample size	As a proficiency study, the study necessarily employed large sample sizes. A total of 252 RNA-seq libraries from 21 batches were generated in eight labs using two library construction protocols (PolyA selection and RiboZero) and two sequencing platforms (Illumina NovaSeq (ILM) and MGI DNBSEQ-T7 (BGI)). In this study, a batch is defined as 12 libraries from a standard sample set, consisting of 12 vials with each representing one of the triplicates of the Quartet RNA reference sample groups, whose library construction and sequencing experiments were conducted simultaneously.
Data exclusions	All data from planed experiments have been included.
Replication	The reference materials were profiled within a batch in a lab in three replicates for each of the four samples (donors).
Randomization	Aliquots of RNA from the same lot were randomly distributed to each center.
Blinding	Each batch of samples distributed was blinded to avoid specific experimental sequences affecting the objective assessment of lab proficiency.

Reporting for specific materials, systems and methods

We require information from authors about some types of materials, experimental systems and methods used in many studies. Here, indicate whether each material, system or method listed is relevant to your study. If you are not sure if a list item applies to your research, read the appropriate section before selecting a response.

Materials & experimental systems

n/a	Involved in the study	
☐	☒	Antibodies
☐	☒	Eukaryotic cell lines
☒	☐	Palaeontology and archaeology
☒	☐	Animals and other organisms
☒	☐	Clinical data
☒	☐	Dual use research of concern

Methods

n/a	Involved in the study	
☒	☐	ChIP-seq
☐	☒	Flow cytometry
☒	☐	MRI-based neuroimaging

Antibodies

Antibodies used	IgA (clone IS11-8E10) (Miltenyi Biotec, cat#: 130-114-002), IgD (clone IA6-2) (BD Biosciences, cat#: 561314), IgG (clone G18-145) (BD Biosciences, cat#: 561296), and IgM (clone G20-127) (BD Biosciences, cat#: 562977).
Validation	PE Mouse Anti-Human IgA (Miltenyi Biotec, cat#: 130-114-002, clone IS11-8E10) was verified by vendor Miltenyi Biotec, including specificity, sensitivity, and fixation. PE-Cy7 Mouse Anti-Human IgD (BD Biosciences, cat#: 561314, clone IA6-2), Alexa Fluor 700 Mouse Anti-Human IgG (BD Biosciences, cat#: 561296, clone G18-145), and Brilliant Violet 605 (BV605) Mouse Anti-Human IgM (BD Biosciences, cat#: 562977, clone G20-127) were validated by our previous study by flow cytometry (Gao, J., Luo, Y., Li, H. et al. Phenomics, 2023, https://doi.org/10.1007/s43657-022-00092-9).

Eukaryotic cell lines

Policy information about cell lines and Sex and Gender in Research

Cell line source(s)	The human immortalized B-lymphoblastoid cell lines of four healthy volunteers from a family Quartet, as part of the Taizhou Longitudinal Study in Taizhou, Jiangsu, China. The sex of the primary cell lines generated from human participants are: F7 (male), M8 (female), D5 (female) and D6 (female).
Authentication	The cell lines have been authenticated by STR profile, karyotype, PCR mycoplasma and sterility testing.
Mycoplasma contamination	No mycoplasma contamination found.
Commonly misidentified lines (See ICLAC register)	No commonly misidentified cell lines were used in this study.

Flow Cytometry

Plots

Confirm that:

☒ The axis labels state the marker and fluorochrome used (e.g. CD4-FITC).

☒ The axis scales are clearly visible. Include numbers along axes only for bottom left plot of group (a 'group' is an analysis of identical markers).

☒ All plots are contour plots with outliers or pseudocolor plots.

☒ A numerical value for number of cells or percentage (with statistics) is provided.

Methodology

Sample preparation	Immortalized B-lymphoblastoid cells were centrifuged at 500× g for 10 min at RT (room temperature). Flick or aspirate to remove supernatant and wash cells with 2 mL PBS at 500× g for 5 min at RT. For the sample stain, 1×10^6 immortalized B-lymphoblastoid cells were resuspended in 100 µl PBS with 2% FBS (FACS buffer) and stained with antibody cocktail for 15 min at RT in the dark. Following surface staining using antibodies, cells were washed twice with 2 mL PBS at 500× g for 5 min at RT. After the final wash, cells were resuspended in 250 µl 1% Paraformaldehyde (PFA).
Instrument	CytoFLEX LX (Beckman Counter)
Software	FlowJo V10.7.2 software (BD Biosciences)
Cell population abundance	This study did not perform sorting.
Gating strategy	For the exclusion of non-single events, cross-check the forward scatter (FSC) signal for its area (A) versus height (H) and width (W) characteristics. Immortalized B-lymphoblastoid cells were gated on the FSC-A versus SSC-A dot plot. Furthermore, IgD+ cells, IgM+ cells, IgG+ cells, and IgA+ cells in immortalized B-lymphoblastoid cell lines were identified based on their expression levels of surface membrane immunoglobulins.

☒ Tick this box to confirm that a figure exemplifying the gating strategy is provided in the Supplementary Information.
